# A Comprehensive Approach to WSN-Based ITS Applications: A Survey

**DOI:** 10.3390/s111110220

**Published:** 2011-10-28

**Authors:** Fernando Losilla, Antonio-Javier Garcia-Sanchez, Felipe Garcia-Sanchez, Joan Garcia-Haro, Zygmunt J. Haas

**Affiliations:** 1 Department of Information and Communication Technologies, Technical University of Cartagena, Campus Muralla del Mar, Cartagena E-30202, Spain; E-Mails: antoniojavier.garcia@upct.es (A.-J.G.-S.); felipe.garcia@upct.es (F.G.-S.); joang.haro@upct.es (J.G.-H.); 2 Wireless Networks Laboratory, School of Electrical and Computer Engineering, Cornell University, Ithaca, NY 14853, USA; E-Mail: haas@ece.cornell.edu

**Keywords:** ITS, WSN, vehicular applications

## Abstract

In order to perform sensing tasks, most current Intelligent Transportation Systems (ITS) rely on expensive sensors, which offer only limited functionality. A more recent trend consists of using Wireless Sensor Networks (WSN) for such purpose, which reduces the required investment and enables the development of new collaborative and intelligent applications that further contribute to improve both driving safety and traffic efficiency. This paper surveys the application of WSNs to such ITS scenarios, tackling the main issues that may arise when developing these systems. The paper is divided into sections which address different matters including vehicle detection and classification as well as the selection of appropriate communication protocols, network architecture, topology and some important design parameters. In addition, in line with the multiplicity of different technologies that take part in ITS, it does not consider WSNs just as stand-alone systems, but also as key components of heterogeneous systems cooperating along with other technologies employed in vehicular scenarios.

## 1. Introduction

The two main concerns with the increasing number of vehicles on the roads are congestion and safety. In the USA alone, congestion accounts for 115 billion dollars in fuel costs [1], with similar figures in other developed countries. Worldwide traffic casualties amount to 1.17 million per year [2]. In this context, Intelligent Transportation Systems (ITS) aim at enhancing transportation efficiency and safety through the use of advanced information processing, communications, control, as well as new electronic technologies.

Sensing the environment is a major aspect of ITS, as well as of other novel applications in future vehicular scenarios. Traditionally these systems have relied on different alternatives [3]. One group frequently employed to detect traffic flows comprises intrusive sensors, including sensors such as inductive loops, magnetometers, pneumatic road tubes and diverse kinds of weigh-in-motion sensors. However, the installation and maintenance of these sensors has important associated costs, since large sections of the road need to be torn up, disrupting traffic flow. Other non-intrusive sensors can also be used, such as video cameras, radars, acoustic arrays and ultrasonic sensors, which can be placed above ground. Their main drawbacks are that they are usually large-sized, power-hungry sensors and may be affected by different environmental conditions. In addition, both intrusive and non-intrusive sensors are expensive and associated with difficult installation, classically requiring wired infrastructures and power lines for energy supply. This leads to the deployment of those sensors only at critical locations, which work independently of each other. The information they produce must be transmitted to distant Traffic Management Centers (TMCs) for centralized data processing, which require the transmission of high amounts of data through expensive communication infrastructures. In general, this results in unacceptable data dissemination delays which limit the utilization of the system for vehicle safety applications requiring a quick response (even real time in most of the cases).

An alternative to these highly centralized solutions is the use of a cooperative approach where processing is performed *in-situ* among distributed devices, enabling faster reaction times. In addition, if this is combined with wireless communications, some of the inconveniences derived from the emplacement of nodes may be alleviated. Vehicular *Ad Hoc* Networks (VANETs) are an example of such combinations [4]. In a VANET, moving vehicles, as well as roadside infrastructure, become nodes of a highly dynamic mobile network that can disseminate relevant information over long distances and collaborate to offer drivers and users improved distributed vehicular services. Nevertheless, VANETs only monitor road conditions opportunistically, that is, when a vehicle is nearby, and their proper behavior is conditioned by the number of vehicles traveling as well as by the penetration rate of such technology into vehicles.

Wireless Sensor Networks (WSNs) are a technology which is becoming more mature and is gaining momentum as one of the enabling technologies for the Future Internet. Therefore, it is being applied ubiquitously and, in particular, to ITS. They consist of medium to large networks of inexpensive wireless sensor nodes capable of sensing, processing and distributing information acquired from the environment through the collaborative effort of nodes [5]. WSNs provide significant advantages both in cost as well as in distributed intelligence. On the one hand, installation and maintenance expenses are reduced because of the use of cheap devices which do not require wiring. Furthermore, distributed intelligence enables the development of diverse real-time traffic safety applications not feasible with centralized solutions.

**Figure 1.**
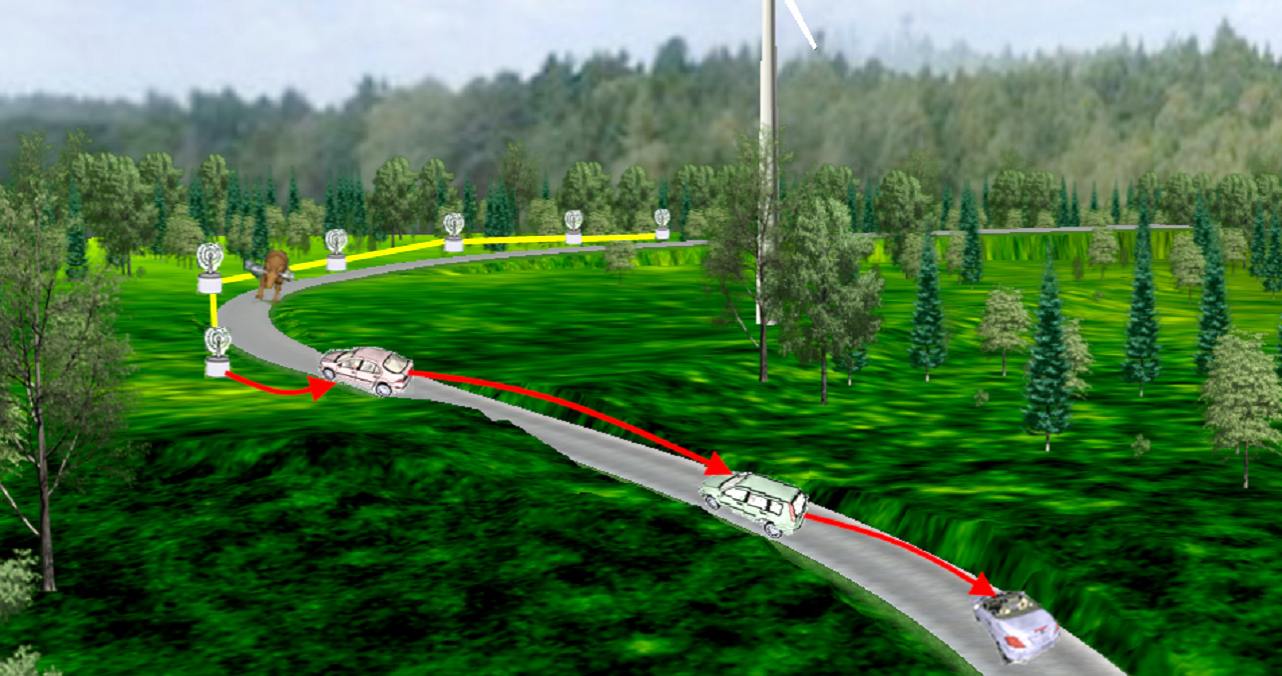
WSN-based ITS application example.

Moreover, WSNs cannot be regarded just as stand-alone systems intended for ITS; on the contrary, they should be considered in the ITS context as additional components of a heterogeneous system, where they cooperate with other technologies such as VANET. Figure 1 illustrates a possible WSN-based application example in which a WSN is employed to detect wildlife on the road and interacts with VANET (or other related technology) equipped vehicles to enhance the driver’s and passengers’ safety and at the same time to avoid, for instance, endangered species fatalities.

Therefore, this survey paper details the fundamental aspects of the design of WSNs for ITS, considering not only WSN independent applications, but also their position in heterogeneous systems. There are other works surveying some specific issues about the application of WSNs to ITS. However, we differentiate from them by adopting a broader approach. In this respect, Tubaishat *et al.* [6] introduced an interesting work about the application of WSNs to ITS, but it readily focuses on estimation algorithms for traffic congestion avoidance, and it does not consider safety or applications that combine different technologies, among others. Mouftah *et al.* [7], in turn, focused their attention on architecture, providing their vision of the architecture of ITS. Our work, conversely, has a more general scope and makes an effort to cover a diversity of closely related technological issues in order to offer the readers and developers a complete state-of-the-art of the actual role and challenges of applying WSNs to ITS. On the other hand, there are other surveys covering the development of heterogeneous ITS systems such as the works presented by Hossain *et al.* [8] and Lee *et al.* [9], however WSN contributions are not tackled in any of them. The former focuses on the challenges of using multiple wireless technologies in a collaborative manner, providing the AHVN (Advanced Heterogeneous Vehicular Network) architecture for the development of applications. The latter is devoted to the so-called Vehicular Sensor Networks (VSN) which are built on top of a VANET by equipping vehicles with onboard sensing devices [10] and, unlike WSN, are not subject to strict resource limitations.

The rest of the paper is organized as follows. In Section 2, the possible applications of WSNs to different traffic domains are presented. Section 3 gives a vision of the network architecture of WSN-based ITS systems which stresses the importance of several factors such as the layout of nodes or the use of heterogeneous devices. Section 4 goes into detail about how vehicle and road state detection is performed by WSN nodes. Section 5 reviews and remarks several crucial design issues which govern the performance of the ITS application. Section 6 deals with the issues related to communication protocols. Finally, Section 7 presents the concluding remarks of this work and introduces relevant open issues which have been identified.

## 2. WSN-Based ITS Applications

WSNs are an interesting alternative to other technologies traditionally used for monitoring. Their use entails low installation and maintenance costs and enables the development of distributed collaborative applications, thus not limiting their functionality to the mere acquisition of data. In addition, WSNs can be used in conjunction with other technologies making more complex applications possible. The functions performed by these applications fall into four different categories: (a) traffic safety, (b) traffic law enforcement, (c) traffic control, and (d) smart parking applications. Some of the most relevant works related to each of these categories are reviewed in Tables 1 and 2 in Appendix according to their main functional properties (more detail about outstanding characteristics of these and other works is given along the paper). In addition, it is also possible that WSNs participate in other applications conducting tasks such as information retrieval (e.g., local services discovery) or entertainment; their contribution to these applications is limited though, as they are in principle less appropriate than other technologies, thus restricting their use to situations where these more suitable technologies are not available.

### 2.1. Traffic Safety Applications

Traffic safety applications deal with the prevention of accidents. In order to fulfill this purpose they make sensor devices work proactively to warn drivers about potentially dangerous situations, such as the presence of obstacles, animals, adverse road conditions (ice or water) and vehicles either stopped (queue-end warning) or driving in the opposite direction (overtaking assistance, wrong-way driving warning). The collaboration among these devices enables to notify drivers of events beyond line-of-sight, thus increasing the available time of response.

There are two ways of approaching these applications, although it could also be possible to develop applications that use a combination of both. In the first approach, upon the detection of the arrival of a vehicle by a static sensor node, the latter activates the subsequent static nodes in order to obtain the condition of the following stretches of the road. This approach has been employed by different applications to support overtaking assistance [11] or animal detection [12], by checking that, correspondingly, there are no vehicles or animals present within a safety zone defined by the application.

The second approach consists of making road information available to nodes before vehicles reach them. This implies that, whenever some data of interest is acquired, it is disseminated within a certain area so that, later, they are gathered by passing vehicles (store & forward scheme). This approach is well suited for the detection of non ephemeral events, such as the occurrence of dangerous road conditions. In this category of applications it is common to find collaboration between WSNs and VANETs, which helps spreading information and prolongs the lifetime of static nodes. As an example, [13] suggests emplacing static nodes at the beginning of each road, which allows all vehicles accessing it to learn in advance about the conditions of the road (previously gathered by other vehicles). A denser deployment is used in [14], in which WSNs monitor the road and VANET disseminates the information either to other vehicles traveling in road segments without WSN infrastructure or to distant static nodes which will warn drivers in the absence of other vehicles.

### 2.2. Traffic Law Enforcement Applications

Traffic law enforcement applications can be considered as a special case of traffic safety applications, since one of the final goals of traffic laws is to increase safety. Currently, traffic law violations are usually detected and put into effect when a police officer or a traffic enforcement vehicle is nearby. WSNs though, offer permanent monitoring of the locations where they are deployed, enabling to automate the process of reporting infractions. Some laws which can be supervised are speeding, illegal parking, going through red traffic lights, unauthorized use of bus lanes or access to restricted or congestion charge areas; yet the first two are those typically included in applications so far. Applications such as [15,16] detect speed limit violations with high precision through the collaboration between adjacent nodes. They rely on cameras triggered upon the detection of the infraction, whose photographs are sent to a Traffic Management Center (TMC) where they are processed and stored. In addition, it is also possible to warn drivers by means of Variable Message Signs (VMS) before proceeding to fining. Illegal parking is also detected by [16] using sensor nodes placed next to curbs which, in turn, after warning through a loudspeaker, activate a camera that takes a picture of the license plate number of the vehicle.

Another application related to traffic law enforcement is post-accident investigation, performed in order to determine responsibilities after an accident. WSNs deployed along the road for a particular purpose get data that is used within a short period of time to fulfill this purpose, e.g., traffic safety. However, it is also possible to permanently store this information and use it later to investigate the causes of the accident (car accident forensics). Although WSN devices are, by nature, constrained devices, they are less and less constrained in storage capacity and can thus hold an important amount of information. Subsequently, this information can be wirelessly gathered by some super-users of the system with special privileges through secure access methods [17], allowing to judge the drivers’ driving style and taking into account the road conditions at the moment of the accident.

### 2.3. Traffic Control Applications

Traffic control involves applications directing vehicles within a road network. These applications consider a road network as a graph composed of intersections (vertices) and road segments (edges), with sensor nodes deployed at and along both of them. Sensor nodes along segments are used to measure the traffic flow in roads, obtaining information such as vehicle density, speed, formation of platoons or distribution of vehicles according to different categories. Sensor nodes at intersections, in turn, are responsible for making the appropriate decisions on how to direct traffic based on the information provided by the sensor nodes along the road. It is also possible to make these decisions in TMCs; however this is a less often explored possibility which implies the transmission of information to a centralized TMC through an external network.

Two different groups of traffic control can be identified. The first group includes traffic guidance applications such as path planning [18] which, due to the size a static road deployment may cover (at low cost), are best suited for urban scenarios. In them WSNs can be used to monitor small to medium size road networks, estimating the time cost of each road segment in order to obtain the optimum path for a specific destination. The second group comprises applications which manage traffic at intersections by means of traffic lights, governing the scheduling of traffic phases (group of directions which are allowed to enter the intersection at a given time). They are based on placing nodes before the traffic lights, possibly one per lane, to find out the number of arrivals at the intersection from each segment [19]. In addition, sensor nodes can also be placed after the traffic lights to obtain the number of departures and, combining both data, infer the queue length at each traffic light [20,21]. These systems require a very small number of deployed nodes, therefore providing very cost-effective solutions. Only if scheduling algorithms relying on the number of vehicles driving towards the intersection (rather than on the number of those waiting) are used, a higher number of sensors are required to forward vehicle detections from distant parts of the road segment.

Traffic control applications need an estimation of the state of the different road segments. Most basic schemes consist of forwarding raw data detected by the sensor nodes towards an intersection or TMC where these data is processed. However, collaborative processing may be applied in order to reduce the amount of information delivered, detecting situations which require traffic diversion in a distributed manner. One such situation is congestion. In [22] a mechanism is used that, on the one hand, reduces the frequency of transmissions by segmenting data into time series of variable length. This length is established by the similarity of sensed data in such a way that only when the traffic state presents significant variations a message is sent. On the other hand, the mechanism reduces the number of transmissions by means of data fusion, making the number of messages delivered increase with the number of nodes of a segment according to a linear progression. This is possible thanks to the use of the Discrete Fourier Transform (DFT) for compression and its property of linearity, which allows a simple and distributed synthesis of the road state from the individual state of every single sensor.

Another situation requiring traffic diversion is the occurrence of accidents. Several methods have been proposed for their detection. A simple scheme is to check if a vehicle did not transmit its condition over a certain period of time. However, in a distributed network, this may imply the interchange of an excessive number of messages between static nodes. Acoustic detectors based on neural networks [23] and vibration sensors in vehicles [24] are more feasible options which provide high accuracy. However, in order to reduce costs and power consumption, a purely collaborative WSN solution such as the shockwave detection algorithm is more appropriate [25]. It is based on the fact that an accident causes two shockwaves in the traffic flow, one of them propagates in the opposite direction of traffic (upstream), forming a queue of stopped or slowing down vehicles, while the other one propagates in the direction of traffic (downstream), decreasing the vehicle density beyond the incident. This method can be seen in practice in [26], where sensor nodes placed along the road estimate traffic volume and detect potential shockwaves which are validated by adjacent sensor nodes.

### 2.4. Smart Parking Applications

The lack of parking spaces in cities is a concern which leads to illegal parking, congestion due to low speed driving and long searching times suffered by drivers. In order to minimize inconvenience to drivers, numerous smart parking systems have been developed which guide drivers to vacant parking spots (PGIS, Parking Guidance Information System) and enable smart payment and reservation options. WSNs are useful for the deployment of smart parking systems as a substitute of more expensive wired sensors. Simpler applications using WSNs may involve detecting the distribution of vacant parking slots throughout several floors by emplacing sensor nodes at the entrance of each floor [27]. However the power of sensor networks comes from the accuracy they provide, allowing to find out about the state of each parking space. There is a considerable number of applications that take benefit from this characteristic [28–30]. In them the WSN is deployed in a grid layout over the parking area, being responsible of the detection of vehicles and leaving other functions such as reservation of parking spaces or guidance to other external subsystems.

Another advantage of WSNs is that they facilitate the development of on-street parking applications. In these applications, unlike off-street parking lots, it is not cost-effective to install additional VMS (Variable Message Signs) or other informative panels in the streets merely for parking purposes. Therefore, on-street parking systems must rely on smart vehicles equipped with On Board Units (OBU), which receive parking information, and with devices for visualization. In order to obtain the location of nearby vacant spaces, vehicles must poll the WSN which will answer with the appropriate information. However, polling from vehicles introduces the problem of mobility, making new routing protocols necessary, which consider that the answers may be delivered to locations different than those where the polling was originated [31].

### 2.5. Factors Influencing Application Design

An ITS application which uses WSNs is affected by several factors which impose or relax the constraints which drive the application design. The most important of them is the limitation of resources of WSN devices. Energy is a scarce resource in WSNs, turning power efficiency into a must. The CPU, in addition, is limited in processing power and its use must be restricted in order to save energy. Something similar happens with wireless communications, offering low rate transfers whose utilization must be reduced in order to improve power efficiency. All of these constraints make WSN a unique technology which requires specialized protocols and algorithms as covered in many different works [5]. In the same way, dedicated software built on top of the popular TinyOS operating system [32], and hardware, for example the well-known sensing platforms TelosB, MicaZ and Mica2 from MEMSIC and their respective sensorboards, which meet the previous constraints, have been developed.

In spite of experiencing these limitations typical of generic WSNs, an ITS application might not always be subject to them. This is due to the existence of reusable road facilities which provide additional energy availability or processing power. Energy availability is given by the access to power lines in some points of the road where traffic lights or VMS are placed. This does not allow powering all sensor nodes but only a few of them; however it may be enough if those nodes are assigned the most demanding tasks. On the contrary, if power lines are not available, solar panels or other renewable power sources as well as energy scavenging techniques are also interesting options.

Regarding processing power, it is possible to integrate WSNs into heterogeneous vehicular technology systems, therefore relying complex processing on more powerful devices such as RSUs (Roadside Units). In addition there are other benefits from the integration with other networking technologies, mainly with VANET and WAN (Wide Area Networks). On the one hand VANETs and WSNs can complement each other. Firstly, they handle different data to a large extent. VANETs use the data gathered from sensors onboard vehicles, which allows obtaining their state. WSN in turn monitors the road itself and may get valuable information prior to the arrival of vehicles. A small overlap exists between the information provided, though. However, this increases robustness. Secondly, they are also complementary in how they propagate information. While VANETs promote information dispersion, WSNs favor keeping information in a static location, enabling schemes which combine both. The combination of VANET and WSN has been referred to by some authors as Hybrid Sensor-Vehicular Network (HSVN) [13,14,33]. On the other hand, the integration with WAN technologies enables transporting the acquired information to distant locations, such as Traffic Management Centers, or to passengers in vehicles by means of cellular networks. As it can be seen, WSNs may be only a part of a global solution composed of different complementary technologies.

The last consideration regards the scenario where the designed system is used. Three generic scenarios may be considered: urban, highway and rural. The choice of the scenario where the application is adopted has an important effect, as it determines the circumstances under which the system operates. Urban scenarios are characterized by the presence of dense grids of streets and intersections as well as medium to high traffic densities. Highways, in turn, present long linear layouts without intersections and peaks of high traffic loads at some points. Finally, rural scenarios have low densities of vehicles and scattered intersections, and unlike the other two scenarios, a low availability of reusable facilities and less frequent maintenance support. Therefore, all of these conditions must be taken into account in the design.

### 2.6. Requirements

The success or failure of a WSN-based ITS application is decided by many factors. As regards the design of the application, there are some of them which, to a greater or lesser extent, must be satisfied. Some of these are as follows:

- **Low cost**. In order to be an attractive alternative to other technologies, a WSN-based system must be cost-effective. This implies reducing the costs of the deployment and maintenance by using as few devices as possible and assigning them the minimum set of functionalities required, reserving more costly functions to a reduced group of nodes.
- **Lifetime**. The deployment of the system infrastructure carries an associated investment. Its benefit will depend on the period of time during which the system is exploited. Given the power restrictions of WSN, this period will be determined by the ability of the network to reduce power consumption and by the use of additional power supply sources.
- **Flexibility and scalability**. A system must be able to adapt to the different situations which are expected under its normal operation. This includes variable traffic conditions and the penetration ratios of smart vehicles in the system. Similarly, it is desirable that a system be versatile enough as not to be restricted to a fixed scenario, making it reusable for different purposes and in different locations. On the other hand, the system must be allowed to grow, either in number of users or in the size of the area it covers. To this extent, growth must be facilitated, making necessary to provide the system with self-organizing capabilities.
- **Robustness and fault tolerance**. Sensor nodes deployed on the road are subject to adverse situations such as the passing of vehicles over them and unfavorable atmospheric conditions which may provoke either the failure of the node itself or its malfunction. This imposes the necessity for means of physical protection. In addition, in case of breakdown of single nodes due to harsh conditions or battery depletion, the overall operation of the network must not be affected, which implies providing the network with redundancy or alternative mechanisms to guarantee connectivity.
- **Appropriate Quality of Service (QoS) provision**. According to the function of the system different QoS parameters must be satisfied including reliability (referring to the correct reception of delivered data at the destination), delay and, in infrequent cases, bandwidth. Safety applications are the most demanding when it comes to reliability and delay, since it is crucial to report any imminent danger well in advance. Other applications are less strict as sporadic losses or delays neither provoke accidents nor probably the malfunction of the system.

## 3. Network Architecture and Topology

**Figure 2.**
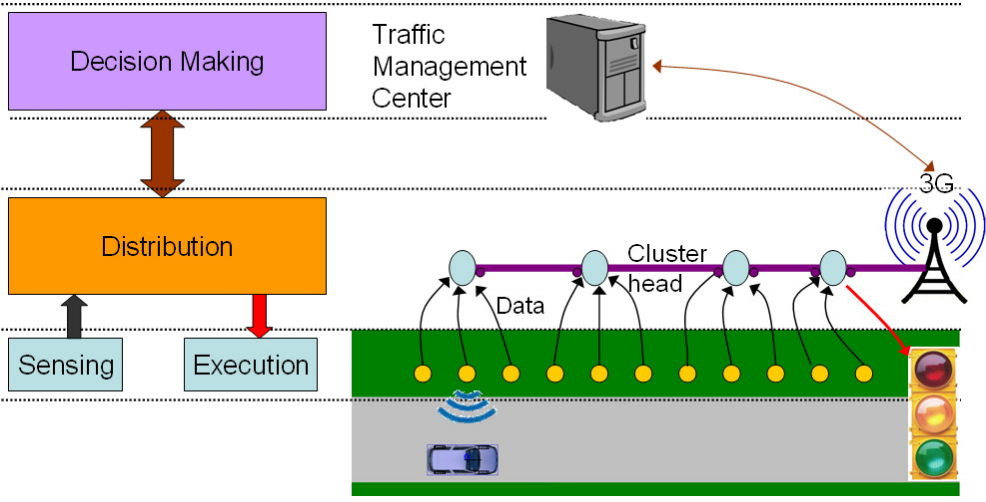
Reference architecture for WSN-based ITS applications.

A distributed WSN-based ITS application accomplishes four different main tasks: (i) acquisition of information, (ii) data distribution, (iii) data processing in order to plan the necessary actions, and finally, (iv) execution of the appropriate actions. Since these tasks may be carried out independently, it can be considered that they correspondingly define four differentiated subsystems which are present in ITS systems, namely, the Sensing subsystem, the Distribution subsystem, the Decision Making subsystem and the Execution subsystem. In this paper these subsystems have been abstracted in a reference architecture depicted in Figure 2, which is inspired by the architectures proposed in [34,35]. It is defined by placing the different subsystems at separate hierarchical levels, where each subsystem may interact with its neighbors at the immediately higher and lower levels. The architecture of a particular system will be based on the allocation of these subsystems into tiers of distributed and potentially heterogeneous devices. This may result in architectures varying from single tiered architectures, where all the devices perform all the tasks of the system, to multi-tiered architectures, in which each tier of devices specializes in the tasks of one or several subsystems as will be described in Section 3.5. The following subsections describe into more detail each of the four subsystems mentioned above. In addition, Table 3 in Appendix summarizes how the reviewed systems implement these subsystems.

### 3.1. Sensing Subsystem

**Figure 3.**
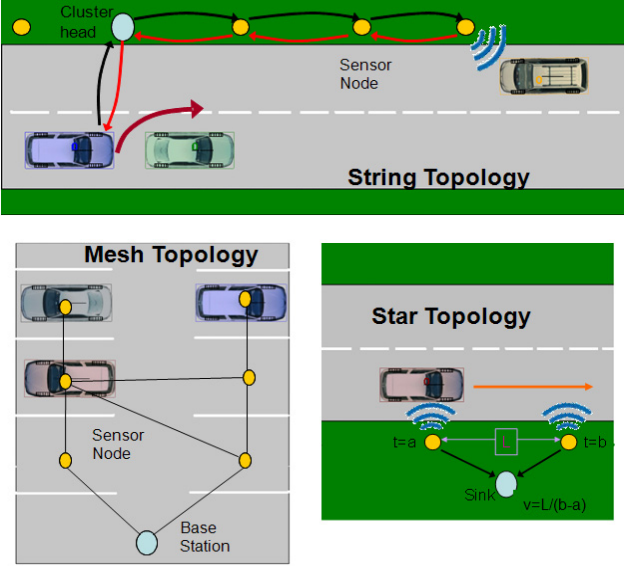
Sensing topologies and examples: **(a)** String topology, overtaking assistance, **(b)** mesh topology, parking lot, **(c)** star topology, speed detection.

The Sensing subsystem is composed of all the devices in charge of acquiring relevant information mainly relative to traffic and road states. In a WSN-based ITS application, not necessarily all the devices use WSN technology, allowing a distribution of tasks among devices using different technologies. However, as regards the acquisition of data, WSNs are the prevailing technology of choice. Consequently, the implantation of the sensing subsystem consists of the deployment of one or several WSNs throughout the observation area (roads or parking lots), which detect vehicles through their sensors and optionally communicate wirelessly with them. After observing how different applications have dealt with the deployment of these WSNs, it was noticed that they follow some basic topological patterns which determine important properties of the system such as lifetime, costs and functionality. WSN nodes are divided into groups following a similar outline, and the deployment consists of a composition, typically homogeneous, of these groups of nodes. Therefore, they can be considered as the building blocks of the sensing subsystem. It should be noted that, in addition, the nodes forming these blocks may propagate information within them, but this should not be confused with the Distribution subsystem. Data propagation in the Sensing subsystem is restricted to local areas, aimed at extracting information from the block/group (towards a sink node) or enabling collaborative processing with nearby nodes. If data dissemination through larger areas is required, for example to transmit information to distant TMCs or to communicate with neighbor blocks, a distribution subsystem is also required. Below, the different topologies which were identified, shown in Figure 3, are introduced:

#### 

##### • Mesh Topology

A mesh topology presents a generic case used in applications concerning many WSN domains. As a result, there are many communication protocols available which are intended for the creation of self-forming and self-healing (fault-tolerant) networks, performing many-to-one and one-to-many communications. Consequently, this topology is adequate for applications which need to deploy their nodes in a grid layout (e.g., parking lots [28]), not necessarily regular, and which deliver their data to a central sink for decision making. However, this topology complicates the development of collaborative applications since the network self-forming mechanisms do not allow to control how nodes establish links between them.

##### • String and Cluster String Topologies

Linear or string based topologies arrange static nodes in a row parallel to the road, leading to an important reduction in the complexity of the routing protocols, since every node only has to decide in which direction to forward. This implies the use of a 1-dimensional geo-routing policy with simple addressing schemes which enables point-to-point communications between nodes, thus simplifying the development of collaborative applications. For example, a node detecting a vehicle can start the collaboration with neighbor nodes just by indicating that it intends to share the event with nodes up to a determined hop count.

Two different alternatives fall into this group of topologies, the uniform string topology [36] and the cluster string one [11]. The difference between them lies in the allocation of tasks among the nodes. In a uniform string topology all the nodes have similar hardware resources and perform comparable tasks regarding vehicle detection and routing. In a clustered string, nodes are grouped into clusters including a more powerful Cluster Head (CH) and several constrained cluster nodes. Through this arrangement it becomes possible for those tasks requiring more powerful hardware or higher power consumption to be accomplished by a small subset of the nodes with additional capabilities; therefore allowing a reduction in costs and prolonging the lifetime of the rest of the network. The typical actions of CHs are the execution of routing tasks to other clusters or subsystems [26], activation of sleeping nodes upon the detection of a vehicle [12] or the communication with vehicles through additional wireless interfaces [12].

##### • Star Topology

In the star topology a few sensor nodes are set around a sink. Its advantages are simplicity and the avoidance of routing schemes, which helps to preserve energy. Two differentiated cases can be considered depending on whether transmissions are performed in a unidirectional [37] or bidirectional way [15]. Unidirectional communications are assumed to deliver information from sensor nodes towards a sink for data analysis. In spite of potentially offering less functionality, they enable additional power savings since sensor nodes are not the destination of any message and, therefore, they do not need to periodically power up their radio and wait for incoming messages. Consequently, these are the most energy-efficient cases of all, especially the former. However, the placement of nodes is limited to the area of coverage of the sink.

##### • Barrier Topology

**Figure 4.**
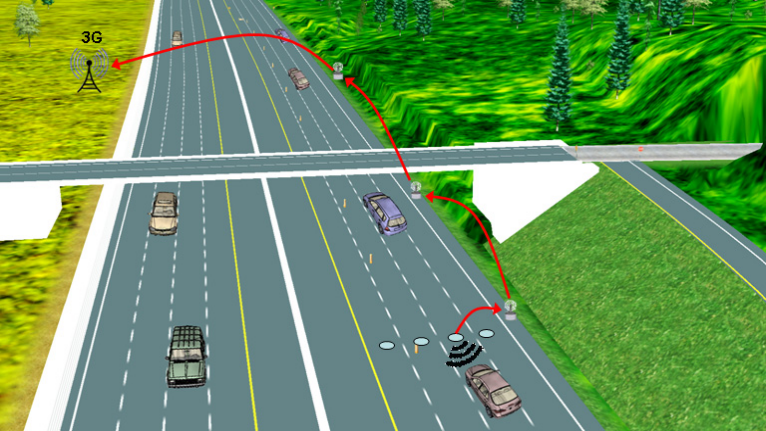
Distribution subsystem based on the availability of cellular networks.

The barrier topology deploys several nodes transversely, one per lane, across the road (see an example in Figure 4). It can be considered as a particular case of star topology, which inherits all of its characteristics. Since it is a very simple topology, its functionality is restricted. However, it allows to obtain the number of vehicles passing a determined point of the road and it is therefore quite useful in applications which need an estimation of the traffic load.

This topology may be employed in a clustered fashion [37]. Each of the sensor nodes at every lane does a simpler function, detecting vehicles and reporting to the cluster head at the roadside (therefore likely to employ only unidirectional communications). Apart from receiving notifications from sensor nodes, the latter manages communications with vehicles as well as with other neighbor clusters through the Distribution subsystem.

##### • Disconnected Nodes (with Mobile Sinks)

A common requisite of all the previous topologies is that there must be connectivity between any static node in the deployment and at least one of its neighbors, which limits the maximum separation between nodes and forces the emplacement of nodes in areas where other neighbor nodes are situated. Using vehicles as mobile sinks can overcome this limitation since disconnected static nodes can send and receive information while a vehicle is nearby. Therefore, they can be placed arbitrarily. This offers several advantages including scalability, since the system may be easily extended by installing a single node at the desired position; robustness, since the malfunction of one node does not affect its neighbors; and finally, as a consequence of not having a maximum separation between nodes, the possibility of covering larger areas at lower cost (at the expense of a low resolution). In contrast, this scheme has some drawbacks, such as requiring that all static nodes be provided with extended capabilities (they all have to sense and communicate with vehicles) or restricting the events about which a node can inform to those happening in its local sensing area, which are transmitted to vehicles when they are close to the phenomenon. The latter can be partially overcome by using communication standards which support transmissions at a longer range than typical WSNs protocols. However, in order to increase the anticipation with which vehicles receive information, the use of a vehicular distribution tier is required [38]. A special class of disconnected networks is the disconnected clustered barriers [33] for multi-lane roads, which combine advantages from clustered barriers with arbitrary emplacement thanks to the use of mobile sinks.

##### • Vehicular Sensing

In addition to obtaining traffic and road information by means of static nodes, it is also possible to use vehicles for this purpose. In this respect, measurements taken from the different sensors installed onboard vehicles, as well as information about the presence of other vehicles, can be transmitted via radio to roadside nodes. Therefore vehicular sensing requires static deployments of one of the arrangements above. The fact that a vehicle itself announces its presence by means of RF transmissions, which henceforth will be denoted as active vehicle detection [14], allows that only vehicles that are able to interact with the system will be detected by the roadside deployment. On the contrary, non-equipped vehicles are disregarded by the system, which is a problem for applications requiring a detailed knowledge of the traffic state.

### 3.2. Distribution Subsystem

The Distribution subsystem is responsible for exchanging information between the different subsystems of an ITS application. In a tiered architecture such as the one in Figure 2, it is placed in a central position, receiving communication requests from all the other subsystems and serving them accordingly. It is in charge of the transmission of sensed data to the Decision Making subsystem and, conversely, of the transmission of commands from the Decision Making subsystem to the Sensing and Execution subsystems [37]. Similarly, it interconnects the different sensing groups of a network (described above). This results in a scalable network created by the composition of these groups. In this respect, the Distribution subsystem can also interconnect physically isolated groups of nodes or vehicles, thus enabling the interconnection of WSN islands deployed in different parts along the road [14] or of clusters of vehicles which other way would form unconnected VANETs [33].

The Distribution subsystem consumes a significant amount of power owing to the requirements imposed on its devices. They are in charge of forwarding every event reported by every node of the Sensing subsystem, which may occur at a high rate. In addition, it must be done under the time constraints defined by the application. This requires very active nodes in order to guarantee information delivery in a timely manner, jeopardizing energy savings. Therefore, the devices used in this subsystem need a more generous power budget than the sensing nodes, thus making additional power sources necessary.

There are different ways of implementing the Distribution subsystem. Although the most obvious is deploying WSNs with external power sources, there are other possibilities. One of them consists of employing vehicular networks to disseminate information. Devices used on the vehicles have no energy constraints since they can be powered by the vehicle’s onboard facilities. In addition, the mobility of vehicles, which is a limiting factor for other kind of applications, helps spreading data throughout vehicles and static nodes along the road, and it relieves static nodes from distribution tasks. The simplest scheme entails using direct transmission between vehicles, *i.e.*, a one-hop network which enables data sharing from a source vehicle to every vehicle approaching it [38,39]. Given the technologies used up to now, this usually translates into mobile WLANs to which other vehicles associate. The second alternative is based on using a multi-hop VANET distribution network [12,40]. This option requires more system resources, but it also facilitates faster data dissemination and scales much better as the number of technologically equipped vehicles grows.

Obviously VANETs exhibit important advantages which make them an alternative to be considered for the Distribution system. However, they also have some drawbacks; the main one is the negative impact of low technology penetration rates on the system performance. If the density of equipped vehicles is not high enough, multihop routes cannot be constructed and connectivity between vehicles is very sporadic. Currently the implantation of VANETs is still in its early stages and full sets of equipped vehicles can only be seen in some research works but rarely in real life. Therefore, alternative solutions with higher penetration ratios are still an interesting option. Clear examples are smartphones, whose number is increasing exponentially, with 297 million sales in 2010, 72.1% more than the previous year [41]. They are not a distribution technology in themselves, but rather devices intended for user interaction. However, they are supported by cellular networks which can distribute information. As a result, gateways to cellular networks such as 3G networks can be included in the system design as the distribution technology [11].

A Distribution subsystem based on cellular networks is an attractive option, not only because of its high availability but also because of its deployment cost. Even gateways are expensive devices which require cellular network interfaces, only a few of them are required to be placed close to cellular Base Stations (BS) and some cheaper nodes which connect the sensing nodes with the gateways must be deployed (see Figure 4). On the other hand, exploitation costs must also be taken into account since cellular operators bill the use of BS. This may lead to another choice either when it is not planned to pay for the use of BS or they are not available, which consists of using IMS (Internet Multimedia Subsystems) [11,42] that provide access to the Distribution System through different radio technologies (2G, 2.5G, 3G, WLAN) regardless of the operator.

### 3.3. Decision Making Subsystem

The Decision Making subsystem (DMS) is in charge of planning the necessary actions in order to achieve the objectives of the system. The tasks which are assigned to this subsystem can be divided into three different groups. The first of them comprises tasks aimed at data storage and preprocessing. It deals with the huge amount of data which arrives at the subsystem, filtering and storing only relevant information and subsequently accessing to it. The second group handles traffic information from different sources and processes it according to the aim of the application. Finally, the third group of tasks is responsible for addressing control commands as well as for managing the network.

The DMS can be executed at different levels. In a top level it can be implemented at centralized TMCs. This implies that all data gathered by the sensing subsystem is sent, via the Distribution subsystem, to the DMS, which must support an asymmetric data flow. The main advantage of this approach is the possibility of performing complex calculations over a great amount of information. Conversely, if only simple processing is to be applied, the DMS can be distributed among the sensor nodes. This enables performing simple collaborative algorithms between neighbor nodes which allow the execution of real-time traffic safety applications. Finally, another solution is the use of smart devices (smartphones, *etc.*) in vehicles, which may receive raw data from road networks and use them, for example, to plan routes.

### 3.4. Execution Subsystem

The Execution subsystem performs the necessary actions which foster changes in the traffic flow according to the objective of the ITS application. It is mainly composed of devices providing visual and acoustic stimuli to drivers, though others aimed at vehicle automation would also pertain to this subsystem. Different equipment may be used. Traffic lights or Variable-Message Signs installed along the roads are attractive options which provide strict control and adaptability to different situations, respectively. They offer the advantage of being widely adopted road infrastructures, suitable for reuse in ITS applications, which help reducing deployment costs. The use of panels attached to the sensing nodes is another possible solution; nevertheless power supply restrictions of unwired nodes limit their application to small panels or informative leds. Finally, the employment of in-vehicle systems offers, on the one hand, the possibility of presenting customized information for every vehicle and, on the other hand, the chance to use acoustic signals and messages that diminish distractions while driving. In addition, the information from the road systems can be integrated into In-Vehicle Infotainment (IVI) systems, for example, for its fusion with digital maps or other information services (transport timetables, weather forecasts, *etc.*). Currently, the considerable and increasing number of smartphones, navigators or tablets paves the way for the adoption of in-vehicle systems. However, there is a growing interest of vehicle manufacturers to incorporate IVI systems in their products as an added value. In this respect, new automobile models from important companies are equipped with systems such as BMW’s iDrive [43], Audi’s MMI [44], Ford’s SYNC [45] or GENIVI Apollo from the GENIVI alliance [46], which aim at facilitating the development of IVI applications.

### 3.5. Network Architecture Classification

The architectures of real ITS systems can be obtained by mapping the subsystems depicted in Figure 2 onto physical tiers of devices. This leads to largely differentiated systems according to the way that it is done. Two top level decisions should be made; firstly, a flat *vs.* a hierarchical network must be selected and, secondly, for the latter case, a homogenous *vs.* heterogeneous use of wireless technologies. The next sections describe different alternatives which can be found in any of these cases.

#### 3.5.1. Flat Networks

In flat networks all the deployed nodes play the same role and therefore perform the same tasks. ITS systems employing them are single-tier systems where a roadside WSN carries out sensing, distribution and, if required, collaborative decision making tasks. Conversely, centralized decision making and execution tasks, due to the limitations of WSNs, are undertaken by other external subsystems. The main advantage of these systems is their simplicity. Nodes with higher complexity are not needed since, on the one hand, it is not necessary to deal with issues related to the interconnection of different technologies and, on the other hand, routing protocols in flat networks are usually simple. However, flat networks have an important disadvantage in what regards scalability. As the number of nodes in a network increases, the gateway node and those around it are subject to an overload which can either degrade the performance (by means of information packet collisions and increased latencies) or force the use of a longer active period in the nodes, thus increasing power consumption.

The lack of scalability of this pattern restricts its use to small areas. As a consequence, it is mostly used for smart parking applications [29,47], in which sensor nodes are installed on every parking slot forming a grid layout which suits the use of generic routing protocols for flat networks [48]. However, it is also possible to find traffic applications using flat networks with string sensing topologies [36]. Their use is feasible in applications aimed at controlling isolated hotspots on the road, not requiring a centralized and distant DMS, for example in overtaking assistance in dangerous locations, since it is only necessary to share events with a few neighbors.

#### 3.5.2. Hierarchical Networks

Unlike flat networks, these networks make use of a hierarchical distribution of tasks among nodes. The network consists of heterogeneous nodes where the most powerful one performs the most demanding tasks and the less powerful nodes are reserved for the less challenging ones, thus saving as much energy as possible. This can be accomplished by using simple schemes. The simplest approach is the one used in many traffic control applications, in which one or few sensor nodes are responsible for traffic detection, while nodes at intersections control traffic lights. However, hierarchical networks are able to offer a more important benefit when they are applied to larger systems.

A well-known class of hierarchical networks is clustered networks, which stand out thanks to their scalability. They are based on grouping nodes into clusters where one of them is selected as the cluster head, which will present the services offered by all cluster nodes to the external devices. This results, as it was stated, in saving costs and energy, since most of the nodes implement only lightweight functions and a few of them, the cluster heads, require a greater investment in more powerful devices and in larger power units and solar panels [11,15]. Scalability is provided by increasing the number of clusters, which does not increase the complexity of each cluster. Consequently, simple routing algorithms can be applied within the cluster, being single hop star topology a feasible option. This can be useful not only for traffic applications [15] but also in parking applications [30], deploying a cluster star network formed by the composition of different groups of nodes with a star topology which transmit to a central base station.

If scalability to wider areas is required there is the possibility that the cluster heads self-organize into a multihop network that delivers information to farther points. The result is a two-tiered system where constrained cluster nodes compose the sensing tier and more powerful cluster heads pertain to the distribution tier, as can be seen in the example shown in Figure 4. This permits sharing road information with distant nodes in order to warn drivers timely as well as communicating with a distant DMS since a tiered architecture allows separating delay sensitive operations (speed measurement, detection of dangerous vehicles) from delay insensitive operations (storage at the DMS). In the latter case, when a distant DMS is present in the system, it can be considered as the third tier of it, in charge of the centralized decision making [34].

#### 3.5.3. Heterogeneous Networks

Heterogeneous systems combine several wireless technologies to facilitate the development of more effective applications. Since every wireless technology offers distinctive advantages and disadvantages, a heterogeneous system seeks to focus on the advantages of a particular technology to compensate the drawbacks of another technology also employed in the final system. For example, WSNs have their main weakness in their constrained use of the scarce available energy. However, this is a minor issue in VANETs. On the contrary, achieving high technology penetration rates in VANETs in order to boost performance is not straightforward, but the installation of WSN nodes on selected roads is a simpler task. One can take advantage of the composition of heterogeneous technologies, which results in the devices of each technology arranged in their own tier, assigning sensing and distribution tasks to either of the tiers.

Two different types of heterogeneous applications can be distinguished. The first are WSN-centric applications, in which a road WSN deployment is complemented by a VANET [12,38] or a cellular network [11,15]. Those applications are quite similar to those described in the preceding sections, but they differ from them in that either VANETs or cellular networks perform the data distribution tasks. This may suppose alleviating static WSN nodes from the burden of long range data forwarding and thus preserving energy. In the case of VANETs, they offer the possibility of propagating information gathered by the WSNs either to other vehicles which are approaching to the sensed area or to distant WSN nodes, possibly detached with the originating nodes. Therefore, vehicles may know the existence of dangerous conditions in advance, as shown in Figure 1 above. The same can be achieved by using cellular networks. However, information follows a different path in order to arrive to smartphones in the vehicles or, thanks to the wide coverage of cellular networks, to distant and centralized TMCs [15]. In this case the distribution tier is not only formed by the cellular network itself, but also by a set of WSN nodes which forward data from the sensing nodes to the nearest base station [11], as illustrated in Figure 4.

**Figure 5.**
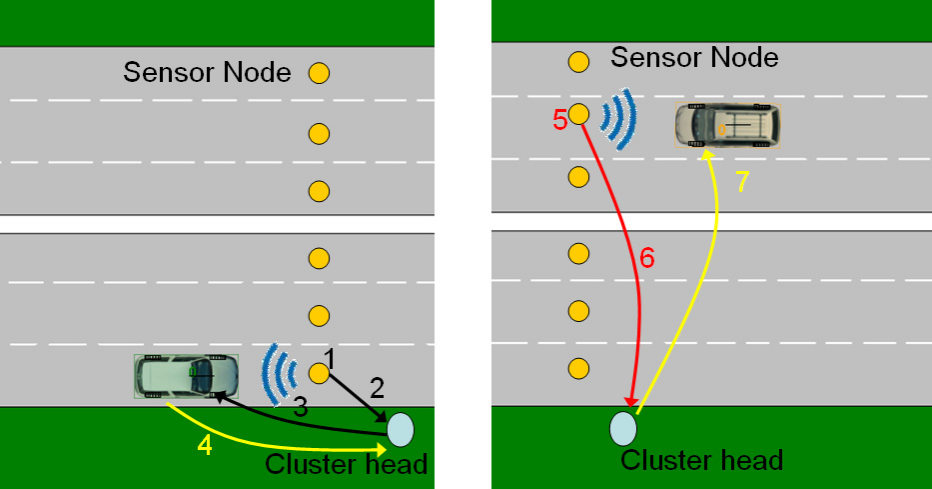
VANET-centric application. Data Interchange between disconnected vehicles: (1) source vehicle detection, (2) notification to Cluster Head, (3) data request to vehicle, (4) data reply (stored at the Cluster Head), (5) destination vehicle detection, (6) notification to Cluster Head, (7) data delivery to destination vehicle.

The other category of heterogeneous applications is the VANET-centric applications. In this category the WSNs are used to improve the performance of existing VANETs [13,33]. They assume that VANETs are established but that they are split into different isolated partitions, which consequently cannot share information. WSNs can solve this problem in an analogous way as RSUs do, *i.e.*, storing information from a partition and delivering it to subsequent partitions when vehicles arrive at the WSN location (as depicted in Figure 5). However, RSUs are scarce and expensive equipment. In contrast, simple WSN islands, ranging from a single node to a barrier of them, are a cheaper solution for those locations where RSUs are not already available or cost-effective.

Applications which involve the use of VANET communication entail the interconnection of two different wireless network technologies. Consequently, they require gateway devices equipped with two wireless interfaces in order to enable the intercommunication between both networks (e.g., portable PCs with WiFi [49] and 802.15.4 [50] or the recently available NEC LinkBird-MX [51] with 802.11p [52] and 802.15.4 interfaces). These are more expensive and power consuming devices, which motivates several saving procedures. Clustering devices are the most common. At the road WSN deployment, assigning gateway functionalities to cluster heads decreases power consumption and costs in the rest of cluster members. Similarly, arranging vehicles into clusters also saves energy, since gateways only need to communicate with the leading vehicle of each cluster which can, in turn, disseminate information within the cluster. Another typical scheme is done by placing the gateways in vehicles, which have greater energy availability [12,14], as opposed to placing them on the roadside [40]. The drawbacks of this option though, are, firstly, that it requires an extra investment since the potential number of vehicles may be greater than the number of static nodes and, secondly, that the interconnection between vehicles and static nodes is accomplished using WSN communication standards which are not specifically designed for vehicular applications, thus offering worst mobility support, radio coverage and throughput. However, standards such as 802.15.4 and B-MAC [53] have been used in applications involving vehicles traveling at low to medium speeds [14,40]. As pointed in [40] the coexistence of both approaches in a same system can be desirable, providing a compromise between costs and achieved performance according to the road operator’s needs.

## 4. Road Sensing

One key feature of WSNs is their ability to acquire information from the environment. WSN nodes are able to obtain raw data from their sensors and process them in order to determine the occurrence of some events. The information that they may provide is very extensive, not constrained to the mere detection of vehicles. For instance, vehicle length and speed can be obtained easily. Besides, more advanced features such as vehicle classification and re-identification can also be accomplished by WSNs. Classification is useful for making statistics of the utilization of roads by different vehicles and it does not boil down to classifying vehicles into a few classes; it is even possible to distinguish between different models of vehicles. Re-identification, in turn, matches the detection of a single vehicle at different locations of the road network, thus enabling vehicle tracking in order to obtain information about travel paths, travel times and origin/destination demands [54]. On the other hand, WSNs have been widely used in environmental monitoring applications; therefore they are a proven solution to detect different situations which may affect driving safety such as adverse atmospheric conditions or the presence of animals or obstacles on the road.

The execution of the sensing tasks can take place under three different scenarios, namely, (i) detecting and monitoring moving vehicles, (ii) detecting stationary vehicles, and (iii) monitoring the road state. The next subsections are devoted to these particular scenarios, which determine the figures of interest that can be measured and how to do it.

### 4.1. Detecting and Monitoring Moving Vehicles

The detection of vehicles traveling on the roads is essential for most traffic safety and control applications. It is characterized by the low time available to detect a moving target, which is bound to the period during which a sensor reports an altered output because of the passing vehicle. In order to properly detect the vehicle at least one measurement must be performed during this period (more measurements for greater reliability), which must be higher than a predefined threshold value. For example, a 4 meters vehicle traveling at a maximum speed of 120 Km/h would require at least a 12 Hz sampling rate, provided that the vehicle produces a noticeable sensing output during all the period where it is close to the sensor; a higher sampling frequency would be required if the excitation of the sensor is not constantly over the chosen threshold value. The selection of the sampling rate is an important issue, since it has a considerable impact on power consumption. Low sampling rates promote the use of duty cycle schemes where both sensor and CPU may sleep while it is not necessary to perform any measurement. Consequently, the lower the sampling rate and the wake-up time of sensors and CPU are, the more energy efficient the system is. Note that there are a considerable number of applications that use cameras for vehicle detection, however we consider it out of the scope of this paper, since they usually involve image processing schemes that exceed the processing capabilities of wireless sensor nodes.

An additional magnitude which can be measured by WSNs is velocity. Although it is possible to obtain the speed of a car using a single node it presents some difficulties according to the method that is employed. A straightforward solution consists of mounting two closely spaced sensors in the same node and computing speed as the quotient between their separation and the difference between detection times. However, in order to provide accurate results, the sampling rate must be very high, thus exhausting power reserves. There are more effective methods for computing speed with a single node, but they require additional information. For example, in order to obtain the speed of vehicles traveling in a platoon, the mean vehicle length is necessary [55]. Using collaborative processing nodes is a more appropriate solution in terms of power consumption since placing sensors at greater distances in separate nodes alleviates the inaccuracy introduced by inadequate sampling rates and minimal displacements of sensors from their optimal positions. At least two nodes separated by several meters are necessary to perform this calculation, though three nodes provide slightly more accurate estimations, since they allow to compensate errors introduced by computing and transmission delays [56].

Provided that the speed is known, it is possible to calculate the length of the vehicles, which can be used as a basic classifier, distinguishing between small, medium and large vehicles (or either between passenger car/minivan, truck, *etc*.). In order to estimate this magnitude several measurements of a same vehicle are required from every single sensor, thus demanding an increase in the sampling rate. However, this must not necessarily increase power consumption since not all deployed nodes need to perform such estimation. Instead it is possible to increase sampling rates only when neighbor nodes detect an incoming vehicle, thus maintaining power consumption low as well as offering accurate length estimations.

**Figure 6.**
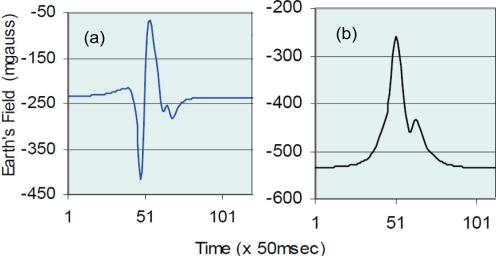
Magnetic signature for: **(a)** longitudinal axis to the direction of movement of a vehicle, **(b)** Z axis. Sensor located at the roadside. From [58], courtesy of Honeywell International, Inc.

The estimation of the vehicle length requires almost no computations but gives a very rough classification. More complex classifications are feasible using the vehicle signatures which sensors may provide. The signature of a vehicle for a particular type of sensor is the variation in time of the sensor output that produces a vehicle traversing the sensing area. An example corresponding to the magnetic signature generated by AMR (Anisotropic Magneto-Resistance) sensors can be seen in Figure 6. Each vehicle class has its own characteristic signature, which enables classifying vehicles that match it. The matching process depends on the sensor used as will be shown in the next subsections.

#### 4.1.1. Sensors

There are several factors to consider before the selection of a proper sensor. These are, among others, the functionality it may provide, the power consumption incurred, the detection range and the need for special encapsulation. The possible applications have been outlined in the previous paragraphs and they determine, to a great extent, the particular sensor employed. The power consumption depends on the selected sensor and, more importantly, on the purpose given to it since it establishes the sampling rate. The range of detection, apart from setting the distance from which vehicles can be detected, also reveals whether a single sensor can perform detections in multiple lanes. However, multi-lane sensing with long range sensors complicates the election of detection thresholds. Conversely, sensors with shorter detection ranges have the advantage of being able to effectively distinguish between traffic in different lanes. The sensitivity to external factors such as temperature, rain or wind may in turn affect the performance of sensors negatively, providing inaccurate measurements which may require filtering or a constant adjustment of the detection criteria. Finally, the use of some sensors introduces additional encapsulation requirements depending on whether the sensor needs to be placed in a part of the road where vehicles may pass, requiring extra physical resistance, or whether it needs to improve its detection capabilities (e.g., use of Fresnel lenses with infrared sensors), *etc.*

##### • Anisotropic Magneto-Resistance (AMR) Sensors

AMR sensors are the most common sensors for detecting vehicles because of their small size and detection properties [57,58]. They are low power and provide accurate detections using low sampling rates of few Hz. The operation of these sensors is based on measuring the variation produced in the Earth’s magnetic field by the ferrous elements of a vehicle, providing outputs for the X, Y and Z magnetic axes. Their range of detection is low, quickly fading out with the distance. Consequently, they are used to detect traffic on a single lane. One of their main drawbacks is that they are very sensitive to the orientation of the sensor and the lateral offset of vehicles with respect to the sensor, which noticeably affects the signature produced by vehicles. In addition, they are also sensitive to temperature which influences the detection threshold values, requiring frequent adjustments.

The signature given by AMR sensors offers valuable information. A signature example can be seen in Figure 6, which corresponds to a sensor placed at the roadside. Besides detecting a vehicle, it allows determining the direction in which it travels inspecting the output provided by the magnetic axis longitudinal to the road. If the falling edge of the signal waveform occurs before the rising edge, the vehicle is traveling in the direction of the axis. Conversely, if the rising edge occurs first, the vehicle is traveling in the opposite direction to the axis orientation.

AMR sensors can be placed either by the roadside or at the center of a lane. Placement at the roadside is more appropriate for detecting vehicles, since the Z axis produces a peak while the vehicle is inline with the sensor, and determining direction of travel. Location at the center of lanes requires to perforate the road in order to install the sensor node flush with the pavement surface and therefore they must withstand vehicles passing over them. To this end, encapsulations based on harsh plastic box as well as epoxy filling may be used as in [57]. Nevertheless, this placement produces more characteristic signatures, with greater precision than the provided by other traditional sensors such as inductive loops. The drawback is that these signatures complicate vehicle detection since it is possible to either report two detections upon the passing of a vehicle or report a single detection for two consecutive vehicles. On the other hand, they are very useful for vehicle classification and re-identification purposes, which can be performed with higher sampling rates, up to 128 Hz. However, the sensitivity to the lateral offsets of vehicles may lead to improper classification if the lateral offset does not correspond to the one with which the reference signature was obtained. A possible solution consists of placing an array of sensors transversally on the lane and selecting, from all the measured signatures, the one that best matches any of the reference signatures [59]. In addition, this also improves the detection of small vehicles like motorcycles, which are only detected when they are closer to the sensors, requiring at least two sensors per lane [55].

The process of matching the signatures requires simple algorithms to enable real-time calculations at the nodes. It is based on obtaining simpler vectors of characteristic parameters from the signatures which can be used to simplify calculations. Representative examples are the Average-Bar and the Hill-Pattern transformations [60]. The former divides the signature into a fixed number of pieces and calculates the average value of each one, thus obtaining an easier but yet valuable simplification. The latter transforms the signature into a sequence of 1, 0 and −1 values by comparing the slope of the signature with certain thresholds. The resulting data vectors can be further compressed by means of techniques such as the Principal Component Analysis (PCA) [61], which produces small vectors that can be effectively processed by the simple classifiers found in sensor nodes [60].

##### • Acoustic Sensors

They can be used to detect vehicles by capturing the engine noise with a microphone. Their main advantage is the long range of detection, much longer than the range of any other sensor presented. It can therefore be used to detect vehicles from further distances as well as in multiple lanes. In addition, the acoustic signature of vehicles can be used to classify them by means of neural networks [62]. However, it has important drawbacks. The high spectral amplitude of the monitored signal requires a very high sampling rate, thus making a duty-cycle scheme unachievable. In addition, acoustic sensors can be interfered by noise originated by different weather conditions such as wind or rain, making them prone to fail and report false positives as well as requiring that the signal be filtered. Finally, they are not appropriate for detecting slow moving vehicles or for high dense traffic situations.

In spite of their shortcomings, they can be used to provide unreliable detections, possibly at low sampling rates, which can be used to wake up other sensors performing more precise detection methods [63]. This is a really attractive option if the acoustic detector is an independent subsystem of the sensor node which, rather than providing measurements upon CPU requests, work autonomously letting the rest of the hardware enter the power saving mode until a vehicle arrives [64].

##### • Passive Infrared (PIR) Sensors

PIR sensors measure the IR radiation emitted by heated bodies, though usually they are commercially available as motion detectors which perform a binary detection of vehicles, causing a loss of detail in signatures. Some sensors though, such as those from the IRA-E700 series do not behave as binary detectors, providing detailed signatures, which can be seen in [65]. PIR sensors and detectors have been frequently used for tracking people but rarely for vehicles [63]. In comparison with other sensors they offer an intermediate range of detection which makes them suitable for multi-lane detection. They can be mounted overhead or at the sideways, though overhead mounting offers better performance. On the other hand, the downsides of PIR devices are that they are affected by atmospheric conditions such as rain, snow or fog; and that the presence of humans and animals triggers false detections. It should be mentioned that PIR sensors usually require Fresnel lenses to improve long distance detection, which can also be used to change the field-of-view of the sensor.

##### • Accelerometers

Accelerometers can sense vibrations of the road caused by passing vehicles. Recently they have been suggested as accurate sensors for detecting and classifying vehicles [66], with potential applications in WSNs [11]. Although their possibilities are yet under study, they are very promising sensors. They provide a signature where output peaks coincide with vehicles axles, which can be used to estimate the number of axles and the wheel-base of a vehicle, and, using this information, its speed (by means of a single sensor). In addition, it is possible to use accelerometers to estimate the lateral offset of vehicles thanks to the different attenuation suffered by the different frequencies [66]. The main weakness of these sensors is that very high sampling rates are required since the signal has a spectral bandwidth of a few KHz, therefore requesting that this sensor be used only on demand, when other less accurate and less energy consuming sensor notifies a potential detection.

### 4.2. Detecting Stationary Vehicles

The detection of stationary vehicles is typically used in intelligent parking applications where the aim is to control which parking spaces are free. Unlike moving vehicles, where the time window for detecting a vehicle is small, stationary vehicles may remain close to the sensor node for a long period of time. This not only improves reliability in detections, since a positive occupancy can be reported after several measurements above a certain threshold, but it also enhances power savings by allowing lower sampling rates and thus more efficient duty-cycles. In addition, the use of adaptive sampling rates, which temporarily increase after the first above-threshold measurement, allows for additional reductions in consumption [30]. Furthermore, the long periods elapsed between samples let the sensor node enter deeper power saving modes, which require relatively long transitions to the active state and are otherwise unfeasible.

Typically the detection is carried out by AMR sensors [27,28,30] despite the fact that there are other alternatives such as the use of light sensors [29]. Cameras and active sensors (ultrasonic, light with laser pointer) have also been tested in some works [27]; however, they show excessive power consumption. In addition, cameras generate a great amount of data to be transmitted and a more complex processing, while ultrasonic and light sensors with laser pointers cannot differentiate between humans and other objects from vehicles, needing to check extra attributes such as speed.

AMR sensors, in the case of parking lots, can be affected by interferences (e.g., vehicles in contiguous parking spaces) which may lead to false positives. If nodes are placed under the vehicles, in the center of the parking spots, the acquired signal from vehicles parked at the place under consideration is noticeably higher than that originated by vehicles in neighbor parking spaces. This, along with the use of properly defined threshold values for the measured signal, can be applied to indentify when a vehicle is currently parked at the sampled space. It should be noted that the measured magnetic signal depends on the type of vehicle above (vehicle elevation, layout, *etc.*); therefore threshold values must be chosen so that they are valid for the different kinds of vehicles using the parking.

According to the open literature, light sensors may be considered either as a good option for detecting stationary vehicles [67] or as totally useless [27]. The huge difference in both of these studies is due to the location of the sensors. These sensors offer inaccurate results when placed in a lateral position (with respect to the vehicles), but provide good outcomes when placed under vehicles, since vehicles block all the incoming light, reporting a positive detection. However, light sensors are quite sensitive to environmental illumination, not being well-suited to work in shadow areas or at night. In [29] some additional reference nodes equipped with light sensors were used to eliminate the effect of environmental light variations. However, how the system behaves in dark conditions was not specified. A positive consideration about these sensors, though, is that they are available with most commercial sensing platforms, thus alleviating the complexity of installing additional hardware.

### 4.3. Monitoring Road Condition

In addition to structural health monitoring of transportation infrastructures such as bridges, typically carried out by WSNs by means of accelerometers [68], WSNs can also be used to examine other aspects related to traffic safety. In this regard, the occurrence of adverse weather conditions is a major concern for traffic safety, accounting for 1.5 million accidents per year in the United States [69]. WSNs can play an important role in reducing the accident rate associated with weather by detecting and reporting in advance different situations affecting the vehicle maneuverability or drivers’ visibility. In contrast to the detection of vehicles, the relatively slow variation in the road conditions facilitates low sampling rates, with the consequent reduction in power consumption.

The sensors employed to this purpose include those typically intended for environmental conditions monitoring with WSNs: PIR and temperature sensors for ice and snow detection, humidity sensors for fog and rain detection, and ambient light sensors. The downside of these sensors in the case of detecting ice and snow is that they can only estimate a probability of occurrence according to other environmental factors. More accurate detection methods commonly imply higher power consumption. However, there are feasible alternatives such as measuring material’s permittivity [70]. This work presents a small sensor which measures the permittivity of the material found between two electrodes at two different frequencies, distinguishing among water, ice and air.

The presence of pedestrians and animals in the immediacy of roads also constitutes a risk. There are many methods for detecting their presence but, if power consumption is the priority, PIR-based detectors are the most appropriate solution. They recognize the presence of moving objects, with a detection range which depends on the size of the targets and whose sampling rates are determined by the motion speeds.

## 5. WSN Application Design

WSNs are a powerful technology to implement and deploy distributed sensing applications. However, during the development of WSN-based ITS applications, a set of design aspects may have a decisive influence on the behavior of the system. This section is aimed at discussing the main design concerns that arise to satisfy good performance and long system lifetime. The unavoidable issues that the designer must face are the placements of the nodes, the duty cycle and the message delivery delay.

### 5.1. Node Placement Constraints

The appropriate placement for the WSN nodes is an important issue dealt by different authors who select the node localization as a function of different constraints such as energy consumption and transmission range. The goal is to find a proper balance between system lifetime, functionality and cost. Focusing on the network lifetime, many authors suggest the use of short range links between nodes, considering that short hops are associated to less transmission power and that several short hops are preferable than a single long hop. However, this assertion, for limited power consumption devices such as those used in WSNs, is examined in several works, and short hops have been proven not to be effective. For instance, in [71] it is shown that, for short hops, the power consumed by the transceiver’s internal circuitry is bigger than that radiated, therefore wasting a considerable amount of energy. The authors provide experimental results of the utilization of commercial devices such as the Mica2 and MicaZ motes, which reveal that variations in the transmission power (and consequently in the transmission range) have little effect on the total power consumption. Many works follow this approach, setting large node separations that still allow small packet losses [12]. Similarly, it is also possible to use long-haul transmissions but relying on smaller node separation, thus allowing transmissions between one node and several of its neighbors. In spite of increasing cost, this adds robustness to the network since multiple paths to a same destination are available. As an example, in [65] this is achieved by selecting the farthest reachable node from all of the closest neighbors as the next-hop (in multi-hop transmissions), and dynamically adjusting the procedure according to packet losses and passive listening of neighbors’ transmissions.

Some works propose the use of cooperative transmission techniques such as cooperative MIMO (Multiple Input, Multiple Output), SIMO (Single Input, Multiple Output) and MISO (Multiple Input, Single Output) in order to reduce power consumption in WSNs and increase system capacity. In [72] it is shown that two cooperative nodes reduce power consumption in comparison with a single node when they transmit to a location which is at a distance greater than 60 m from them. The employment of more cooperative nodes improves power consumption when transmitting to locations at even larger distances. For example, three cooperative nodes consume less power than two nodes for transmissions to distances greater than 80 m, four nodes consume less than three for distances greater than 140 m, and so on. WSN-based ITS applications are good candidates to benefit from cooperative transmission techniques since there are many situations where it is possible to find nodes close enough to cooperate. This may happen when nodes are deployed at both sides of a road or at intersections. The intersections deployment case has been studied in [73], considering the deployment of nodes at road signs in crossroads. The purpose is to achieve low power communications between crossroads (applying MIMO techniques) and between crossroads and vehicles (MISO, SIMO), with the latter being equipped with directive antennas in order to establish communication with crossroads even at further distances.

Another consideration about the placement of nodes is related to the position of nodes within the road. For single lane and two-lane roads, nodes may be deployed either by the roadside or at the center of the lanes. The differences between both options regarding their respective performance were mentioned above, in Section 4. For multi-lane roads, placement at the center of the lane is required. Emplacing nodes at the center of the lanes, where vehicles may pass, implies that these nodes require special encapsulations and protections and that the nodes have to be installed aligned with the pavement. This leads to a reduction in transmission distances because of the ground effect suffered by the antennas of the nodes, which affects its radiation pattern in the directions parallel to the ground. This issue motivates the use of clustered barrier topologies (described in Section 3). An example of these can be found in [37], where two types of nodes are used in the deployment: (i) nodes located on the center of the lanes, with a transmission range of a few meters, and (ii) nodes on the roadside transmitting to neighbors at a distance of 50 meters. The second type of nodes can be placed at more elevated locations to reduce the ground effect. Transmissions occur from nodes at the lanes to their closest roadside node and, then, the messages are forwarded by the roadside string topology.

The location of parking lots’ nodes is different to that stated in previous paragraphs. Node separations are necessarily small, as they correspond to the separation between parking spaces. However, in order to transmit to data sinks or cluster heads, these separations may be larger. The problem, in this particular case, stems from the disturbance that metallic vehicles cause to the communications. As it was written in Section 4, nodes must be placed at the center of the parking spaces in order to achieve proper detection. However, this implies that whenever a vehicle parks in the space under consideration, it may block the signal sent and received by the node beneath. Therefore, the transmission ranges are considerably reduced, as shown in [28]. In the worst case, for a transmission between two nodes at ground level that are covered by two parked vehicles, only lateral communications are possible at a maximum distance ranging from 2 to 4 meters. If only one of the nodes is covered by a vehicle, transmissions are reliable up to 5 meters, erratic up to 10 meters and impossible for greater distances. In some cases, if transmission ranges are not large enough, the use of intermediate relay nodes may be necessary. This has been studied in [74], proposing an algorithm to optimize the emplacement of relay nodes in order to minimize power consumption.

### 5.2. Duty Cycle

The best way to save energy in WSNs is maintaining nodes at non-operational power saving modes as much as it may be possible. The power consumption of the most consuming components of a node such as the CPU, radio and sensors varies significantly depending on their particular operational state. For example, the current drawn from the CPU in the sleep mode ranges between 1 μA and 50 μA depending on the processor technology, which is three orders of magnitude lower than under normal operation. Similarly, the radio consumes a comparable amount of current, for example for a MICAz sensor node, it ranges from less than 1 μA in the sleep mode to several mA in the operational mode [56]. In addition, for most commercial devices, the power spent in the receiving mode is comparable to the one consumed while transmitting. Consequently, rather than reducing the number of transmissions, it is more beneficial to reduce the period during which the radio is active (listening to the channel). Power savings for all these components are usually accomplished by means of a technique known as “duty cycling”, which manages the activity periods of the nodes. It is aimed at dividing the working period of a node into two main parts, namely: (i) the active period, where the components perform their operation, and (ii) the sleep period, where they remain inactive. The ratio between the time the system is active *versus* the total time under consideration (active plus sleep periods) is called duty cycle. This concept is usually applied to the operation of the radio equipment. However, it can be extended to the sensor devices as well, being possible to use either the same duty cycle or a different one for both. In any case, a small duty cycle helps saving large amounts of energy and so it extends node and network lifetimes, as it allows not only the radio and sensors, but also the CPU which drives their operation, to periodically enter the sleep mode. On the other hand, small duty cycles may jeopardize the proper behavior of the system. Hence, an appropriate scheduling of the duty cycle is critical in order to avoid the loss of events of interest and to offer adequate QoS guarantees, especially for real-time safety applications.

The duty cycle design determines the duration of both active and sleep periods. A first consideration regards the duration of the entire sleep/wake-up cycle, which must find a compromise between power consumption and responsiveness. This duration has a lower limit imposed by the wake-up times of the components of the node. Under this threshold the node is not power-efficient since the energy consumed in frequent activations of components is higher than that saved during the sleep period. Processors and radio transceivers are typically the most restrictive components to these regards, with wake-up times ranging between a few μs and several ms depending on the technology employed. However, some sensors may impose higher and therefore more restrictive times. Conversely, the desired level of system responsiveness defines the upper limit of the whole cycle and it is directly related to the duration of the sleep state that is described next.

The limitations imposed on the sleep period depend mainly on its impact on the vehicle detection by the sensors and on the event propagation done by the radio. Regarding vehicle detection, the sleep period cannot be larger than the time spent by a vehicle passing near a node, otherwise there is a high probability of missing the event. This period is, therefore, directly related to the speed of vehicles and the detection range of sensors. However, there are some works that propose operational modes which assume that a vehicle must not necessarily be detected by a single node since it can be detected by subsequent nodes [36]. Concerning the radio, long sleep periods may negatively affect the dissemination of events by introducing additional delays in each message transmission. In an extreme situation, it could even cause the speed at which events are propagated to be lower than the speed of vehicles. This may occur if sleep periods are longer than the time required for a vehicle to move from one node to the next one (assuming one packet forwarding per cycle), which would be a major issue for many applications.

The active period, in turn, must be designed to assure the proper transmission of information. This does not only include the transmission of the information itself by merely reserving a transmission time according to the maximum number of messages that can be transmitted per period and their length. It also includes time deviations, which must be taken into account, too. On the one hand, this implies reserving additional time to handle the synchronization variations that the system may be subject to. On the other hand, if the system allows packet retransmissions (due to packet collisions in the channel access or to transmission errors), the active period must be extended to accommodate them. The extent of this non-ideal operation depends on the selected MAC protocol (Section 6.1). In addition, the use of routing protocols may also increase the active period duration and consequently the power consumption, because it may become necessary for nodes to receive and propagate additional messages from their neighbors, thus having to reserve additional fully operational time for the reception of messages.

Given the differing frequency of events of vehicle traffic, the use of a fixed schedule for the duty cycle is not always the best option. In this respect, there are some works which vary the duty cycle according to parameters related to the traffic state and safety; in particular, they focus on adjusting the duty cycle of the sensors but not the one followed by the radio. For example, in [56] the speed of vehicles is used as the parameter governing the duty cycle. In [36], in turn, several operational schemes are proposed in which the duty cycle of the nodes is controlled by a master node. The objective is the detection of vehicles in order to report potential vehicle collisions on the road. Upon the detection of a vehicle from the master node, all subsequent nodes along the road are woken up. The risk of taking this approach is that if the master node does not detect a vehicle, none of the subsequent nodes will do it. As a result, master nodes are supposed to be more powerful nodes (in terms of energy) with less restrictive duty cycles to ensure vehicle detection. In a similar way, the loss of the wake-up messages also jeopardizes the detection of vehicles by the nodes. Therefore the authors of that work suggest the use of schemes based on the combination of wake-up messages with random wake-up for the nodes. In spite of this, it is still possible that some nodes remain asleep. Some works instead, focus on assuring communication in order to avoid this situation. For example, in [12] a similar scheme based on the wake up of nodes is implemented, but packets are sent by using guaranteed access to the radio channel and packet acknowledgements and retransmissions.

### 5.3. End-to-End Latency

Some applications are subject to strict delay requirements. In general, applications where vehicles need to obtain some information about the road are more sensitive to the delay. The maximum delay that is allowed is determined by the nature and dynamics of the sensed data, which ranges from slow changing road conditions to very fast vehicles suddenly entering a critical area. The impact of exceeding this maximum delay depends on the purpose of the designed application, being especially critical for safety applications. In order to obtain the actual delay to which information delivery is subject to, it is important to take into account the operation mechanism of the application, since it determines the number of times a packet is forwarded in a multihop network. These mechanisms were classified in Section 2 for safety applications, though the proposed classification can be extended to other applications not intended for traffic safety where vehicles or road deployed devices are the destination of the information gathered by sensor nodes. It considers two different groups of applications, those in which safety information is already deposited in road deployed nodes prior to the arrival of vehicles, and those in which the arrival of vehicles triggers a polling/activation mechanism to subsequent nodes along the road. Mainly, the difference between both types of applications with respect to the delay is that the former are subject to a one-way delay due to the forwarding of messages from the source node to the destination. However, the latter suffer a two-way delay as they require query messages to be transmitted in the forward direction and responses alerting about potentially hazardous situations to be sent back in the opposite direction. Consequently, these applications following a query/response fashion further complicate the design of the system.

Apart from the number of times a packet is forwarded, it is also important to consider what the additional delay introduced by each packet forwarding is. The sources of this delay include the time spent by the sender to construct and send the information packet, the time elapsed in gaining access to the radio channel, the propagation time over the air and the time employed by the receiver to process the message. Among these the most important by far is the access to the channel, due to the scheduling of the radio. It prevents nodes from transmitting packets just when they are generated, making them wait until the radio is active and the channel idle. This leads to the introduction of a cumulative delay which increases as each node forwards the packet under consideration in a multihop network. As it will be described in the next paragraphs, this delay is quite dependent on the medium access control mechanism (MAC) in use. In those works taking the delay into account two different approaches have been found, distinguishing between scheduled contention based schemes [75] and TDMA (Time Division Multiple Access) schemes.

Contention based schemes such as CSMA (Carrier Sense Multiple Access) do not schedule transmissions but rather make nodes contend for the access to the channel. However, in order to achieve low power consumption, methods based on CSMA are normally used in conjunction with duty-cycling schemes, using a common schedule among different nodes. An advantage of using such a combination is that, despite the fact that transmissions are subject to a relatively long initial delay while waiting for the start of the active period, it is possible that a packet be forwarded through several hops during a single active period if this period is properly dimensioned. In addition, if the application requires two-way communications, the delay may be barely affected by the forwarding of responses, since nodes may remain in the active state until these are transmitted back. This can be seen in [11], where in a single active period a short query packet travels through 16 different nodes in 10 ms. In this work a 1% duty cycle is used, which does not jeopardize power consumption, and having a sleep time for the radio of 0.99 seconds, the maximum delay is not excessive. On the other hand, the drawback of using CSMA is that packet collisions among nodes trying to transmit may occur. This makes the delay unpredictable since competition for accessing the medium in case of collisions would prevent from forwarding all packets within a single active period, thus prolonging the waiting time during additional sleep periods. A possible solution to this problem would be limiting the number of nodes capable of transmitting packets, *i.e.*, imposing that within a certain area only one node is able to generate new packets to transmit, while the remaining nodes are devoted to packet forwarding, thus avoiding collisions. In this context there are proposals such as [65], where only a master node is allowed to generate queries to subsequent nodes. In that work, the master node makes successive queries directed to each of the nodes arranged in a string pattern and then waits for a response. It is however more reasonable, for most applications, to query all nodes at once by forwarding a single query packet since it achieves much smaller delays.

In opposition to CSMA, TDMA-based MAC protocols guarantee that only one node can access to the channel at a time. In them, time is divided into periodic frames, and frames, in turn, into time slots. During the duration of a time slot only one of the nodes that share the medium is allowed to transmit. Similarly, destination nodes must be in the reception mode only during that slot. Planning the slot assignment among nodes is one of the issues when deploying TDMA networks. However, in the case of road-oriented ITS applications, this task is simplified, given the small number of neighbors per node. On the other hand, because TDMA-based protocols are collision-free, their use leads to more predictable delays than CSMA since the access to the medium is guaranteed (the only uncertainty is due to transmission errors which may imply retransmissions or result in information losses). The expected delay, despite the fact that it can be calculated a priori, depends on the final assignment of slots to nodes, and it may lead to longer delays than CSMA if it is not properly done. A simple method of assigning time slots which optimizes information delivery time in one direction is presented in [12], which allocates consecutive transmission slots to neighbor nodes. In this way, whenever a query packet is transmitted by a node, its subsequent neighbor will forward it in the next time slot of the same frame, thus achieving very fast data dissemination. This technique has however no effects on applications with two-way communication delay. In these the replies may suffer a long delay, corresponding to the length of a whole frame minus one time slot, since any packet to be retransmitted should wait for the time slot assigned to the neighbor node in the next frame; therefore all the time savings done in the forward direction are lost in the backward direction. As a consequence, the authors propose the use of VANETs when possible for the dissemination of warning messages in the backward direction. An additional concern related to TDMA MAC in applications with two-way communications is that nodes are required to stay in the reception mode twice as much as with one-way propagation, thus increasing power consumption. This is due to the fact that nodes need to receive messages from preceding nodes in both directions. It should be noted that in CSMA, for the same situation, while the active time of the nodes does not increase, the probability of collisions does. Nevertheless, if nodes increase the duration of the active state after receiving query packets as proposed in [11], the system can handle more easily this problem.

## 6. Communications

This section deals with the concept related to the design and selection of appropriate communication protocols for a WSN-based ITS system. Basically, this means dealing with Medium Access Control (Section 6.1) and routing (Section 6.2) protocols. In addition, it should also be noted that in some situations it could be desirable to have specific protocols to handle the interaction between different networks. For example, in [33], where the interchange of images between static road nodes and vehicles is envisaged, a communication protocol was designed in order to allow fast transmissions of data during the short interval a vehicle is under the transmission range of a static sensor node. In the same line, in [76] the case that the required information could not be delivered on time by a single static node is considered, making subsequent nodes in road sending the remaining information. Similarly, if the vehicle turns at an intersection, the static deployment based on the reception of acknowledge packets from the vehicle, detect the change of road and assigns the tasks of sending information to a new node.

### 6.1. Medium Access Control

The Medium Access Control (MAC) layer provides link-level data addressing and access control mechanisms to the physical medium (radio) shared by nodes in a multi-point network. The design of MAC protocols for WSN is different from that for other wireless networks since it is mainly focused on power conservation, which implies finding a trade-off between different metrics such as latency and throughput in order to extend the network lifetime. In the scientific literature, many MAC protocols suited for WSNs can be found as well as survey works according to different purposes, including [77], where real-time MAC protocols are considered, and [75], which deals with energy-aware protocols.

Conversely, MAC protocols for VANETs must provide high reliability and low delay without having to deal with the limitations inherent to WSN. To this aim, several works study how the selection of the MAC concerns the communications among vehicles, including [78], which describes the main features of IEEE 802.11 standards applied to the VANET environment, and [79,80], which provide two alternative classifications for MAC protocols. Among the existing protocols and standards, it should be emphasized the recently release of the IEEE 802.11p [52] standard which is intended to offer an efficient communication in the V2V (vehicle-to-vehicle) as well as in the V2I (vehicle-to-infrastructure) scenarios.

The focus of this work is on the WSN tier of the system. The approach which has been taken to review how both existing (general purpose) and specifically designed MAC protocols apply to ITS scenarios follows the classification of protocols proposed in [75] because the different categories considered have an important impact on the performance of the system. According to the classification, on a top level we can differentiate between (i) unscheduled or random protocols, where nodes operate independently; (ii) scheduled protocols, which organize communications in an ordered way, with radio transceivers following a coordinated scheduling of their duty cycles; and (iii) hybrid protocols, which combine different scheduled and unscheduled techniques. In addition, sub-classifications for each one are also presented, where it is interesting to remark that scheduled protocols may be divided into contention and contention-less based protocols.

Unscheduled protocols are popular in the WSN domain because they do not require neither clock synchronization among nodes nor global topology information. Nevertheless, these protocols may suffer higher rates of packet collisions since they usually do not provide any means for avoiding them, apart from carrier sensing. In an ITS scenario they provide two important advantages: (a) dynamic node joining, which is very useful in ITS since it facilitates communication with moving vehicles, and (b) adaptability to changes in topology. The latter have proven to be very important in some specific applications such as those requiring WSN deployments in parking lots. In them, the arrival of vehicles and its associated distortion on communications (see Section 5.1) may provoke the loss of existing links as well as worsening synchronization as shown in [28], favoring the adoption of unscheduled protocols. Within these protocols, preamble based ones are the most commonly found. In them, a sender probes the receiver with repetitive sequences of bits until the latter awakes. These sequences are denoted as preambles in the B-MAC protocol [53], which is currently selected for many ITS works [40,67,81]. In addition the B-MAC protocol is sometimes inadvertently used by developers because many commercial sensor nodes advertising 802.15.4 compliant transceivers actually use B-MAC by default. The use of this protocol in ITS scenarios has two additional consequences. First, end-to-end latencies are high for data dissemination even with short preambles since prior to each forwarding a preamble must be transmitted. Second, applications with high vehicle traffic imply the generation of numerous packets and a considerable amount of energy wasted in the transmission of their preambles. Thus B-MAC is more appropriate for applications involving low vehicle traffic densities such as those found in rural environments or some parking lots. In addition this protocol has demonstrated its feasibility to communicate with vehicles at the typical speeds of these scenarios [40] if this feature is needed.

Regarding scheduled protocols, these are more complex protocols which usually need control messages in order to maintain synchronization and coordinate the radio transceivers active period according to different strategies. This is not an important problem if these protocols are used in road static deployments. However, it may become a serious issue if these protocols are considered for the vehicle to road infrastructure communications, given the limited communication time with travelling vehicles and possible packet losses.

In contention based scheduled protocols nodes basically follow a common active/sleep schedule of their transceivers, using CSMA during their active period. Representative examples can be found in the reviewed literature. S-MAC [82], currently applied to ITS in [11], synchronizes local areas or clusters, implying that in applications with communication between adjacent clusters the data dissemination delay increases and that border nodes must maintain more than one schedule. In addition, the A-MAC protocol [83], used in [31] for a street parking application, outstands for its ability for adjusting the duty cycles of nodes according to their remaining energy in such a way that it can guarantee a predetermined network lifetime.

Scheduled contention-less protocols comprise those based on TDMA and slot reservation. On the one hand, they offer the advantage of avoiding collisions, with the consequent improvement in performance. On the other hand, they also have some drawbacks such as their lack of scalability and adaptability due to the difficulty of introducing new nodes in the layout as well as the need for strict synchronization in order to align slot boundaries; the particular layout of road deployments though, alleviates some of these restrictions. This is the main reason why it is possible to find different protocols specifically designed for ITS systems [12,76,84]. These protocols exploit the layout of roads, characterized by the presence of strings of road nodes with few neighbors, to assign (and reutilize) time slots to non-conflicting nodes in the road (*i.e.* distant nodes). Similarly they assume a hierarchical architecture with more powerful master nodes which manage the rest of nodes. PEDAMACS [84,85] in particular, used in [20,26,55], relies on a master node with a larger communication range which enables it to simultaneously transmit to all the managed nodes via one-hop communications with the purpose of scheduling communications and simultaneously achieving network synchronization; data transmissions from the managed nodes, conversely, occur in the opposite direction in a multi-hop manner in order to save energy. The protocol presented in [76], in turn, stands out for allocating some time slots exclusively for the communication between road nodes and vehicles.

Another commonly used protocol is IEEE 802.15.4 [50], employed in different works such as [15,35,86], which allows low power communications and has a coverage range similar to other wireless technologies such as Wi-Fi and may be used both for contention based (CSMA) or contention-less (TDMA) communications. It should be remarked that this protocol was not designed with mobility in mind, therefore it is well suited for WSN road deployments but it has several problems with mobile nodes related to association with 802.15.4 coordinators and synchronization [87], as a consequence its suitability for vehicle to roadside communication should be studied more in-depth.

### 6.2. Routing

Many routing protocols have been designed specifically for WSN where energy saving is the primary concern. The research about these protocols is reflected in different general purpose surveys [48,88,89], as well as in more specific ones, such as [90] which deals with real-time protocols. In general, the selection of a routing protocol is closely related to the network architecture of the system and the topology of its sensing subsystem. The former fact is reflected on [48], which proposes a protocol classification based on the underlying network structure, and which is quite similar to the one proposed in Section 3.5, differentiating between flat, hierarchical, and location-based routing protocols.

Routing in VANET, on the contrary, has to deal with the mobility of vehicles which produces constant changes in the network topology. Surveys on this topic may be found in the literature in [91] and [92]. In the latter, the protocols are divided into: *Unicast* where a vehicle creates a source-to-destination routing path via wireless multi-hop transmission or carry-and-forward techniques; *Multicast*, *where* packets are delivered from a single source to a multicast group members by using multi-hop communication; *Geocast,* where a packet is transmitted to a specific geographic region, and finally, *Broadcast,* where a vehicle sends messages to all other vehicles in its coverage range.

In an analogous way to the preceding section, the classification introduced in [48] (flat/hierarchical/location-based) will be used to guide this subsection in order to explain the different contributions to routing done by the authors of the reviewed works.

Routing in flat networks is totally dependent on the selected topology for the sensing subsystem. This also applies to intra-cluster routing in clustered networks. From the presented topologies in Section 3.1, only the mesh and the string topologies are able to perform multi-hop routing. The rest of topologies instead rely on direct point to point communications with a sink node. It is remarkable the considerable power savings that these topologies may achieve if they use unidirectional communications, *i.e.*, the detector nodes merely detect events and notify the sink without receiving commands from it, given that detectors’ transceivers may be in sleep mode for extended periods of time, until the detection of an event. Mesh topologies, mostly used in smart parking applications, can be routed according to many different fashions and protocols which are out of the scope of this paper. However an important consideration must be made. The aforementioned disruption of communication links caused by vehicles parked over sensor nodes deployed in parking lots produces bursty packet losses, with high time correlation but low spatial correlation. As a consequence mechanisms based on acknowledgements and retransmissions are not appropriate for reliability. This is studied in [28], where authors instead propose the use of multi-path transmissions to assure that packets are correctly received by the sink, more specifically they use a selective flooding algorithm. Finally, string topologies support simple linear forwarding. In them a node just forwards either to the preceding node (upstream) or to the subsequent node (downstream), despite some works consider transmission to non-adjacent nodes (e.g., two hops away) in order to find a compromise between efficiency and robustness [65]. In addition it simplifies the development of protocols for neighbor discovery [36], and therefore self-organization, or data fussion [22,35], which allows traffic control applications receiving more sporadic updates about the traffic state rather than continuous messages indicating the presence of a single vehicle, possibly reported several times by different nodes.

As the size of the network grows hierarchical networks and routing protocols become necessary. In most cases routing is performed from multiple data sources to a single base station (bottom-up). This is also the case in WSN-based ITS applications in spite of the fact that there are many applications dealing with top-down or peer to peer routing [16,37] or considering them as future work, as will be explained later. This data propagation scheme allows for simple routing, either linear routing between Cluster Heads [37], possibly using cluster members as intermediary nodes [26], or constructing a routing tree between them [30,37] in which each node forwards every message to its parent. In both of the approaches data fusion tasks, if needed, are performed by the cluster heads.

A top-down routing of information between a base station and other nodes is useful in order to issue commands to the latter ones (e.g., turning on warning systems, taking a photograph, *etc.*). Multicast is an easy option if a routing tree exists, making each parent transmit to all its child nodes. Transmission to specific nodes, in turn, has been handled according to different ways. For example in [37] it has been accomplished by means of a source routing scheme, which forces a more powerful base station to manage a routing path to each destination but avoids intermediary nodes such as cluster heads from managing routing tables. In S3 [16] a level based static addressing scheme has been used to simplify routing, which in addition enables peer to peer and table-less routing. It is based on the construction of a tree and the address allocation to each node according to its position in the tree. This is currently used in the S3 system in order to allow that sensor nodes measuring speed trigger the capture of a photograph by a nearby camera-equipped sensor node without the need of previously routing to the base station, since the address of the camera node provides all the required routing information.

Other works focus on the auto-configuration of the network. For instance, DGS [15] uses dynamic addressing for this purpose, automatically assigning 16-bit network address during the construction of the routing tree. In [26], incremental growth of the network is provided by means of an auto-discovery mechanism based on local broadcasts. In this work nodes deployed at different roads may communicate thanks to a hierarchy of controllers/cluster heads which act as simple routers. The discovery takes place iteratively: each node, regardless of its level in the hierarchy, discovers its closest neighbors in both directions as well as its preceding controller in the hierarchy. The routing mechanism allows sending messages to other nodes on the same road or to the preceding controller if the destination is not on the same road. This controller may, in turn, route the message to its presiding, adjacent or subordinate controllers in order to reach the destination of the message.

Finally, the last routing scheme covers the location-based routing protocols, which use position information to route data to the desired regions. This scheme is quite frequent in VANET by means of the so-called geocast protocols. To this regards there are WSN-based proposals that, rather than implementing geographical routing on the WSN side, add geographic information to the data they gather and rely on VANET to propagate them to the appropriate region [40]. Other proposals in which vehicles extract information from the WSN using a polling mechanism (e.g., street parking availability checking) rely on location-based protocols on the WSN side [31,93] since, due to the mobility of vehicles, the response may need to be delivered to a different location from the one where the data was originally requested. In these protocols once the requested data has been obtained by the WSN, the response is routed according to the position, speed and direction of the vehicle. In [31] each node forwarding the response selects the next hop according to the current time and expected destination of the vehicle, which periodically updates its mobility information. In [93], in turn, the vehicle itself estimates its positions at different future times and communicates them to the WSN nodes when requesting data.

## 7. Conclusions and Open Issues

This paper presents a survey of application of WSNs to ITS. As it has been shown, WSN is a technology which may have a relevant role to ITS, enabling cost-effective and accurate solutions with a wide variety of applications in driving safety and traffic control as well as in parking management. Its contribution is not merely about sensing the environment but about making advanced collaborative ITS applications possible. In this respect, in addition to processing data in centralized TMCs, WSNs can be used to process information *in situ*, reducing data distribution costs and offering a fast response to critical events.

The plethora of innovative possibilities that WSNs confer can be further extended if they are complemented with the joint use of other technologies such as VANET or WAN networks, allowing different data dissemination schemes. Therefore, WSNs may take part in heterogeneous ITS in which every adopted technology is used for the purpose it best serves. In spite of this heterogeneity, the design of WSNs for ITS is driven by the same basic premises that any efficient WSN application must satisfy. This is achieved by finding a balance between, on the one hand, low power operation and processing complexity, and, on the other hand, QoS assurance, which is typically accomplished by applying effective duty cycling schemes and task assignment among the nodes. However, these applications differ from other WSNs applications in the additional restrictions that ITS systems impose and in the opportunities they offer. This is mainly due to the mobility of vehicles, subject to relatively high speeds and motion bound by roads. This affects a great number of issues including the detection and estimation of significant features from vehicles, the placement of nodes and the design of routing algorithms, the latter implying a simplification in the development of collaborative applications. In addition, since vehicles pass by sensor nodes in a sequential way, it is possible to develop predictive and adaptive applications as well which correspondingly may learn about the arrival of a vehicle in advance or adapt their action prior to the arrival, thus allowing both power savings and accurate operation.

There are however, some concerns and opportunities in the ITS scenario which either have not been addressed yet or have been only partially tackled. One such example is security, which is only rarely included in WSN-based ITS works, and for which there are really few specific research works [17]. Currently, the adoption of existing ITS tailored security mechanisms poses an important increase in energy consumption which makes them unfeasible, thus requiring some improvement. In addition, there is a focus on securing the information originated by roadside WSN nodes, not considering the case where these nodes obtain information from vehicles. In this respect there is a wide range of potentially vulnerable situations not well covered which can be inferred from security works in VANETs [94].

Synchronization is another issue to be considered. In spite of the existence of a great number of works which consider synchronization, there are few specific solutions for the WSN-based ITS case [85]. Nevertheless, vehicular scenarios enable interesting possibilities involving moving vehicles. A very common problem found in synchronization is the cumulative error introduced in each hop when nodes try to synchronize along a multi-hop path. However, in vehicular scenarios, one can take advantage of the moving vehicles to synchronize a whole string of roadside sensor nodes, one by one, using simpler mechanisms and obtaining more accurate results.

Multi-purpose systems, in turn, remain as preliminary proposals in several works but without being extensively addressed yet. These systems use a same deployment in order to perform different goals, e.g., traffic safety and traffic control, thus making them more flexible and valuable. This implies obtaining and dealing with different kind of information for every purpose (safety warnings, traffic load, hotspots’ pictures, *etc.*), though it is possible to deduce some of this information (such as traffic load) from the interchange of previous messages, thus reducing communications overhead. In addition, this may require the establishment of priorities for the different types of network information flows.

Several works consider the use of the WSN-oriented 802.15.4 standard for the Vehicle to Infrastructure communication (and vice versa). As it was mentioned, this standard is not suited for mobility. Only a preliminary evaluation of the effect of the vehicle speed on the link quality has been conducted [14], stating that up to 70 Km/h there are packet losses but these are not important. However, the association process of mobile devices to static coordinators at the roadside is not considered, which is required by the standard. The main goal of the association is searching for the radio channel in which the network is operating prior to joining it. This process may take up to several minutes, being therefore unacceptable in most ITS scenarios [87]. A straightforward solution is to force all sensor nodes to operate in a predefined channel and modify the protocol operation to automatically select that channel without performing any search. However, this prevents from selecting between different channels in order to avoid interferences from, for instance, nearby Wi-Fi networks. Consequently, there is a need to investigate effective methods for the channel selection. This has been done in other scenarios considering mobility [95], but efforts are still required to find proper solutions in the vehicular scenario.

A reasonable alternative though is the use of appropriate VANET standards for Vehicle to Infrastructure communications, in spite of being less power efficient. Nevertheless, the development of vehicular applications has been affected until now by the unavailability in the market of devices capable of doing this. With the recent publication of the IEEE 802.11p standard, this is no longer a major concern, leading to a massive appearance of compliant devices which may act as OBUs and RSUs [96]. In addition, this equipment fosters the integration with other wireless technologies by means of pluggable interfaces, which may support WSN standards such as IEEE 802.15.4.

Similarly, there are other existing pieces of hardware which may solve some of the problems that have been presented throughout the paper and whose use has not been reported yet. One of these problems is the relatively limited transmission range of 802.15.4 in comparison with standards such as 802.11p. In this case, there are 802.15.4 compliant devices such as Xbee Pro [97] which could be used in order to extend the transmission range to distances greater than 3 Km, thus enabling either a higher separation between nodes or more time to alert vehicles. In case of using these devices, it should be noted that the design constraints change since, instead of minimizing the duty cycle, the main goal would be minimizing the number of packets transmitted as they consume 3 to 4 times more energy than their reception mode. Something similar happens when trying to solve the ground effect found when sensors are placed on the road surface or in small holes in it. For the latter case however, instead of more powerful devices, arrays of antennas could be considered in order to modify the radiation pattern to provide its maximum in the plane parallel to the road.

Finally, another open issue regards the sensors and methods employed for the detection of vehicles. In this respect, in currently WSN-based employed systems, the CPU has an important implication in detection, managing the operation of the other components. This increases power consumption, since the CPU must be turned on for every data sampling. A less consuming alternative implies the use of autonomous low-power vehicle detectors, which rather than being activated by other subsystems, are in charge of taking them out from the lowest power operation modes only when a vehicle arrives. However, to the authors’ knowledge, this has not been successfully implemented yet in current ITS systems. On the other hand, apart from the need for reducing power consumption of individual sensor nodes, it is also a necessary to increase its functionality. Such achievement may require the use of new sensors different than the popular AMR magnetic ones. A clear example is the use of accelerometers, which are expected to allow a single sensor node to perform complex functions such as classification and re-identification, as well as others which previously involved several sensor nodes, such as speed and lane position estimation.

## Appendix

**Table 1.** Functional description of selected WSN-based ITS traffic safety and law enforcement applications.

**System**	**Main Purpose ***	**Data Source**	**Strategy Upon Data/Danger Detection ****	**Strategy Upon Vehicle Detection ****	**Data Consumer (Acts or Warn Driver)**
iRoad [11]	Overtaking assistance*	Roadside WSN	2. Warn upstream nodes of presence of vehicles 3. Send to WAN/IMS	1. Activation of downstream nodes (upon start of overtaking move)	Smartphones running iRide app, leds on the road
Work by Qin *et al.* [12]	Traffic safety (presence of deers, ice...)	Roadside WSN	2. Warning upstream nodes, propagation by WSN & VANET	1. Activation of downstream nodes	Equipped vehicles
SNMS [38]	Traffic safety	Roadside WSN	1. Store in local node	2. Forward: † (a) VANET (b) mobile WLAN	Equipped vehicles
Work by Weingärtner *et al.* [14]	Monitoring road condition	Roadway WSN	1. Store (dangerous) road conditions in neighbor WSN nodes	2. Forwarding to incoming vehicles, VANET dissemination to distant vehicles & WSN nodes	Equipped vehicles
Work by Tripp *et al.* [33]	Traffic safety	equipped vehicles	1. Vehicles store information of the road (e.g., photograph of next intersection)	2. WSN detect vehicles, triggering information interchange with RSU	Equipped vehicles
SNTISS [34]	General purpose	Roadside WSN	(a) Static monitoring: report to remote server. (b) Dynamic monitoring (e.g., tracking): share with neighbors for collaboration		Remote Server
Work by Sung *et al.* [37]	Collision warning	Roadside/Roadway WSN	–	1. Collaborative vehicle speed measurement and routing to Base Station	Display near base station warn vehicles moving in the opposite direction
DGS [15]	Speed, weather monitoring	Roadside WSN	2a. Speed: warn/photo 1b. Weather: Report via WAN	1a. Calculate speed	Speed: VMS, camera Weather: Remote Server
S3 [16]	Illegal parking & speed control	2 Roadway WSN: speed & parking	–	Speed: Collaborative speed measurement Parking: Notify positive detections of vehicles in forbidden area	Loudspeaker/VMS, camera, infraction reporting to server
Work by Festag *et al.*[40]	Safety (road conditions), post-accident investigation	Roadside WSN	1. Store dangerous road conditions in WSN	2. Notification of danger to vehicles, VANET dissemination (only to vehicles)	Equipped vehicles, forensic team with tinyPEDS [98] client

* Main purpose of the system considered or the one detailed in the paper, use for other purposes may be feasible.

**: Order of execution denoted by preceding numbers.

† Alternative options considered in the paper.

**Table 2.** Functional description of selected WSN-based ITS traffic control & smart parking applications.

**System**	**Main Purpose**	**Data Source**	**Strategy for Vehicle Monitoring**	**Data Consumer**
Work by Yousef *et al.* [21]	Traffic light control	multi-lane WSN	Count vehicle arrivals and departures	Traffic control Box connected to sink
WITS [35]	Traffic control at intersections	roadside WSN, Equipped vehicles	WSN propagate speed, location provided by vehicles to intersection nodes	Traffic control equipment Connected to intersection nodes
Work by Tubaishat *et al.* [20]	Traffic light control	Multi-lane WSN	WSN transmits number of incoming vehicles to intersection	Intersection node decides traffic policy and inform nodes at lights
SPARK [29]	Smart parking	Sensor nodes at parking spaces	Periodic monitoring of parking spaces and direct forwarding to unique WSN sink	Guidance & status displays, Remote clients
Work by Benson *et al.* [28]	Smart parking	Sensor nodes at parking spaces	Periodic monitoring of parking spaces and routing to base station	Remote client
PGS [30]	Parking lot guidance	Sensor nodes at parking spaces	Periodic monitoring of parking spaces and routing to base station	VMS
Work by Tacconi *et al.* [31]	Street parking/parking lot	Sensor nodes at parking spaces	Check parking availability upon vehicle request	Equipped vehicle

**Table 3.** Structural description of selected WSN-based ITS applications.

**System**	**Architecture**	**Sensing Topology**	**Sensing Technology**	**WSN Comm. Protocols (MAC, routing)**	**Gateway Vehicle/Road**	**Distribution**
iRoad [11]	Heterogeneous, clustered	String	AMR, accelerom.	SMAC, linear forwarding	None	WSN, 3G
Work by Qin *et al.* [12]	Heterogeneous, clustered	String	PIR (WiEye)	TDMA-based, linear forwarding	802.15.4	WSN (to CH), VANET
SNMS [38]	Heterogeneous	Disconnected nodes	–	–	Bluetooth	VANET/mobile WLAN
Work by Weingärtner *et al.* [14]	Heterogeneous, clustered	String	road condition sensors & Active vehicle detection	802.15.4	802.15.4, dynamic assignment of GW/CH	Short range: WSN Long range: VANET
Work by Tripp *et al.* [33]	Heterogeneous	Barrier	Vehicular sensors	–	802.11b	VANET
SNTISS [34]	Clustered	Application dependent: (a) Star (b) Mesh	multi-sensor	Intra-cluster: 916 MHz CH to Server: 802.15.4	None	WSN
Work by Sung *et al.* [37]	Clustered	Star/Barrier (x2) according to number of lanes	AMR (HMC1021)	Unspecified MAC, routing to BS: tree-based routing from BS: source routing	None	WSN
DGS [15]	Heterogeneous, clustered	Star	AMR, Weather Station	802.15.4, dynamic addressing/routing	None	Cellular network
S3 [16]	Hierarchical	Star	AMR	802.15.4, multi-level (tree) routing based on static addressing	None	Internet (form WSN to Server)
Work by Festag *et al.* [40]	Heterogeneous, clustered	Any (unspecified)	Environmental sensors (temp, humidity, light)	B-MAC, tinyLUNAR	B-MAC (proposed 802.11p alternative)	VANET
Work by Yousef *et al.* [21]	Hierarchical	Barrier (x2)	COTS (unespecified)	TDMA-based, Point-to-Point	None	None
WITS [35]	Hierarchical	String	Vehicular, Active detection	802.15.4, linear forwarding with data fusion	802.15.4	WSN
Work by Tubaishat *et al.* [20]	Hierarchical	Barrier (x2)	AMR (HMC1051Z)	PEDAMACS	None	WSN
System	Architecture	Sensing Topology	Sensing Technology	WSN Comm. protocols (MAC, routing)	Gateway vehicle/Road	Distribution
SPARK [29]	Flat	Star	Light (included in MTS310 sensorboard)	unspecified MAC, Point-to-Point	None	WiFi/BT/Ethernet (DMS to other subsystems)
Work by Benson *et al*. [28]	Flat	Mesh	Magnetic (Speake FGM-3)	Framelets, selective flooding	None	WSN
PGS [30]	Clustered	Star	AMR	802.15.4, tree-based routing	None	WSN
Work by Tacconi *et al.* [31]	Hierarchical	Mesh with designated roadside GWs	Unspecified (simulation)	A-MAC, geographic routing (to estimated interception GW)	A-MAC	WSN
